# Stereoselectivity in spontaneous assembly of rolled incommensurate carbon bilayers

**DOI:** 10.1038/s41467-021-21889-8

**Published:** 2021-03-10

**Authors:** Taisuke Matsuno, Yutaro Ohtomo, Maki Someya, Hiroyuki Isobe

**Affiliations:** Department of Chemistry, The University of Tokyo, Bunkyo-ku, Tokyo Japan

**Keywords:** Stereochemistry, Supramolecular chemistry, Carbon nanotubes and fullerenes

## Abstract

The periodicity of two-dimensional entities can be manipulated by their stacking assembly, and incommensurate stacks of bilayers are attracting considerable interest in materials science. Stereoisomerism in incommensurate bilayers was first noted with incommensurate double-wall carbon nanotubes composed of helical carbon networks, but the lack of structural information hampered the chemical understanding such as the stereoselectivity during bilayer formation. In this study, we construct a finite molecular version of incommensurate carbon bilayers by assembling two helical cylindrical molecules in solution. An outer cylindrical molecule is designed to encapsulate a small-bore helical cylindrical molecule, and the spontaneous assembly of coaxial complexes proceeds in a stereoselective manner in solution with a preference for heterohelical combinations over diastereomeric, homohelical combinations. The rational design of incommensurate bilayers for material applications may be facilitated by the design and development of molecular versions with discrete structures with atomic precision.

## Abstract (web summary)

Incommensurate pairing is a type of stereoisomerism, observed in carbon bilayers, that arises from the twisted orientations of the graphitic layers. Here, the authors create a finite molecular version of an incommensurate carbon bilayer in the form of two concentrically assembled cylindrical molecules.

## Introduction

**Fig. 1:**
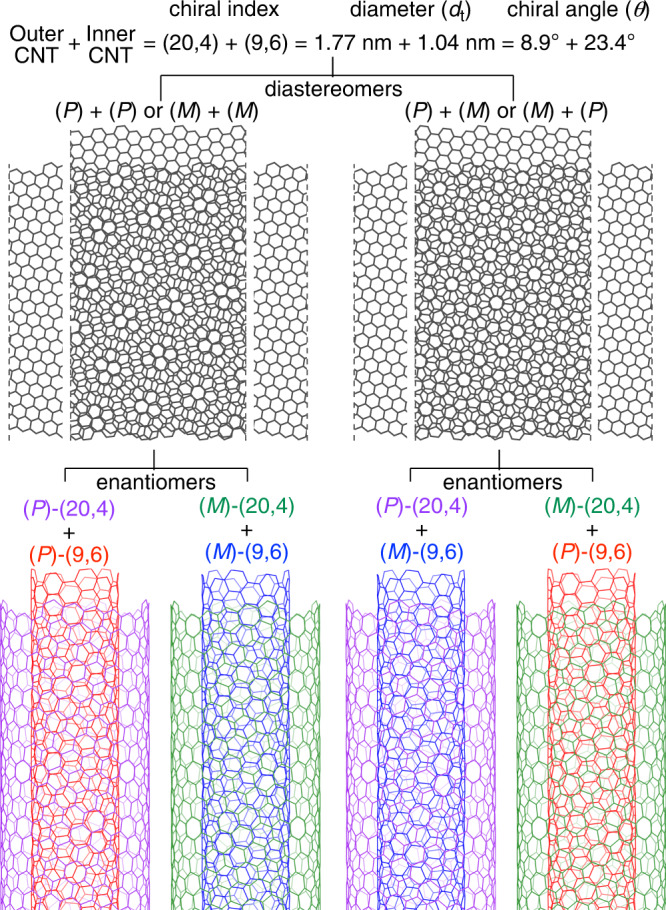
Stereoisomerism of i-DWNT. An example of a combination of two helical CNTs, i.e., (20,4)- and (9,6)-CNTs, is shown. Combinations of two carbon layers in twisted orientations give rise to two diastereomers, each of them existing as a pair of enantiomers.

When two graphitic sheets are stacked to form carbon bilayers, there emerges an interesting example of stereoisomerism in the majority of cases called incommensurate pairs. The stereoisomerism originates from the twisted orientations of two graphitic layers, and the induced chirality and periodicity result in intriguing electronic properties^1–3^ Owing to the dimensionality reduction via facial discrimination, this unique type of stereoisomerism is easier to comprehend by rolling the carbon bilayers in the form of incommensurate double-wall carbon nanotubes (i-DWNTs)^4–6^. Thus, in the case of i-DWNT, stereoisomerism appears in a pair of helical carbon nanotubes (CNTs) comprising an incommensurate set of (*n*_1_,*m*_1_)- and (*n*_2_,*m*_2_)-chiral indices with the relationship of *m*_1_/*n*_1_ ≠ *m*_2_/*n*_2_. For instance, when (20,4)-CNT encapsulates (9,6)-CNT, two diastereomers emerge from facial combinations of the bilayer stacks of helical carbon networks having unidentical chiral angles (Fig. 1). Each of the diastereomeric orientations of the carbon bilayers gives rise to a set of enantiomer pairs; therefore, the coaxial assembly of incommensurate pairs of CNTs results in four stereoisomers of (*P*)/(*P*), (*M*)/(*M*), (*P*)/(*M*) and (*M*)/(*P*) for the double-wall combination of (20,4)- and (9,6)-CNTs^7^. Although such stereoisomerism in carbon bilayers has long attracted attention, particularly in the fields of physics and materials science, the structures have not been well understood and realised in a discrete form for molecular entities.

Here we show that incommensurate pairs of finite CNT molecules, i.e., i-DWNT complexes, can be assembled in a spontaneous manner through the design of tight-fitting cylindrical molecules. An outer helical CNT molecule has been designed to encapsulate a small-bore helical CNT molecule, and the coaxial i-DWNT structure has been determined via spectroscopic and crystallographic analyses. Unexpectedly, the spontaneous assembly is controlled not only by cylinder diameters but also by graphitic helicity, and the helical handedness of the inner CNT molecule is discriminated by the outer CNT molecule. For the assembly of i-DWNT complexes, heterohelical inner/outer combinations of (*P*)/(*M*) and (*M*)/(*P*) are preferred over homohelical combinations of (*P*)/(*P*) and (*M*)/(*M*). Interestingly, this is opposite of the suggestions made previously for infinite CNTs. We anticipate that the stereoselectivity observed with the incommensurate pairs of finite CNT molecules may facilitate the molecular design of chiral moiré patterns for materials applications^8,9^.

## Results

### Design and synthesis of outer CNT molecules

**Fig. 2:**
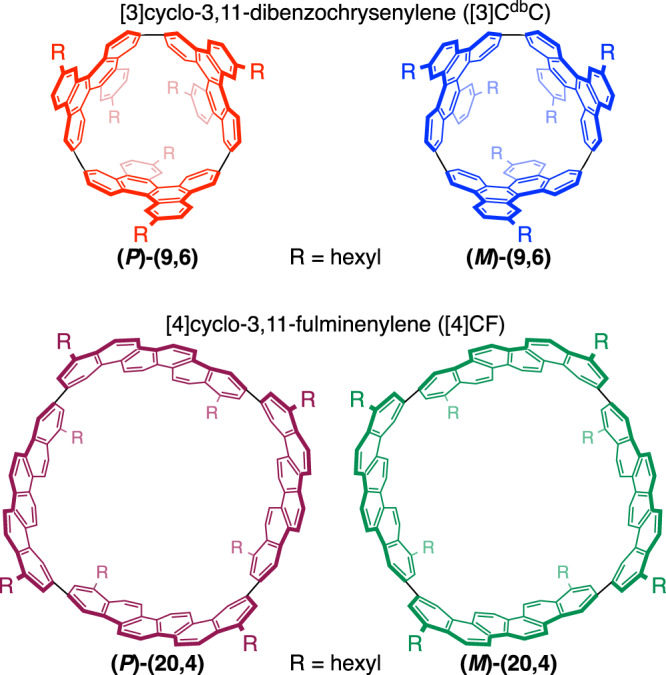
Helical CNT molecules. Chemical structures of small-bore [3]C^db^C molecules and large-bore [4]CF molecules.

The i-DWNT complexes were designed and synthesised by using [*n*]phenacenes as common bases of cylindrical molecules that have different diameters. With chrysene ([4]phenacene) panels, the first example of helical CNT segments was previously prepared in the form of belt-persistent [4]cyclochrysenylenes ([4]CC)^10,11^, and with a contorted chrysene panel of dibenzochrysene, a small-bore helical CNT molecule was synthesised in the form of [3]cyclo-3,11-dibenzochrysenylene ([3]C^db^C, Fig. 2)^12^. We envisioned that an outer CNT molecule may be designed to encapsulate the small-bore [3]C^db^C by using larger [*n*]phenacene panels and sought possible structures by using a simple yet effective 2D-mapping method^13,14^. The small-bore helical CNT segments were (*P*)- and (*M*)-(9,6)-[3]C^db^C, which are hereafter denoted as **(*P*)-(9,6)** and **(*M*)-(9,6)**. The small-bore [3]C^db^C molecule has a geometric diameter (*d*_t_) of 1.04 nm, and, considering the interlayer distance of carbon bilayers as 0.34–0.38 nm^15,16^, the ideal geometric diameter for outer cylindrical molecules should be in the range of 1.72–1.80 nm. By mapping [*n*]phenacenes on a graphitic sheet in various orientations and combinations (Supplementary Fig. 1), we were delighted to find that an isomer of [4]cyclo-3,11-fulminenylene ([4]CF) at a chiral index of (20,4) showed a geometric diameter of 1.77 nm. Thus, the isomers of [4]CF with *D*_4_-symmetric (*P*)- and (*M*)-(20,4)-[4]CF, which are hereafter denoted as **(*P*)-(20,4)** and **(*M*)-(20,4)**, became our target as an outer cylindrical molecule for i-DWNT complexes.

**Fig. 3:**
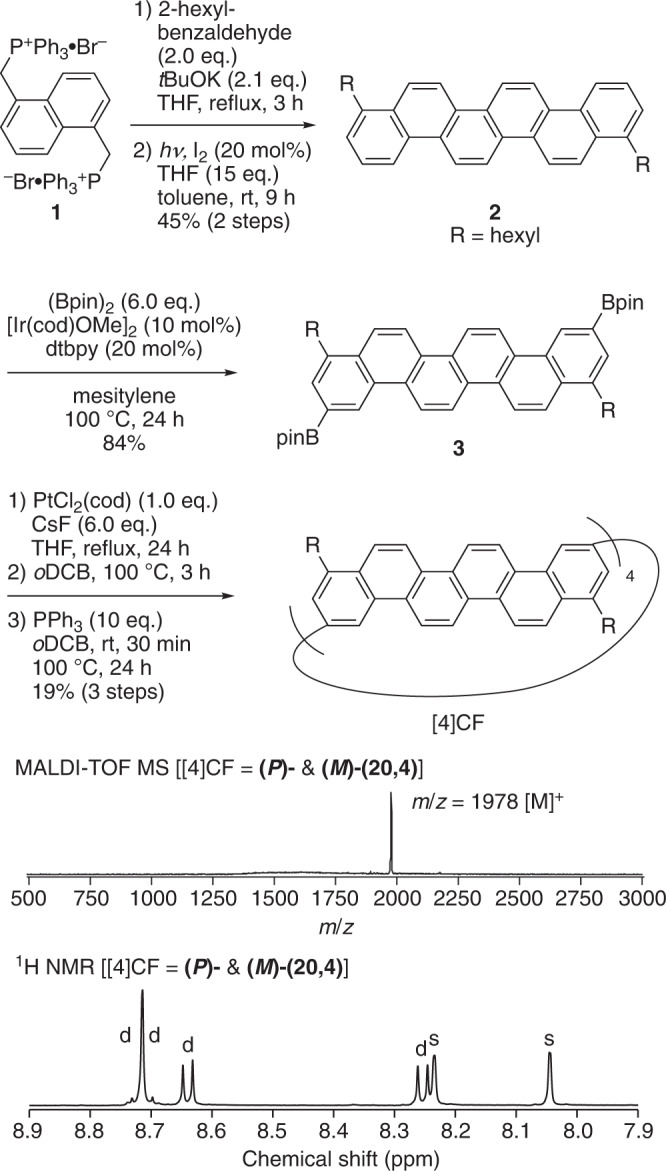
Synthesis of [4]CF [(*P*)- and (*M*)-(20,4)]. Bpin = 4,4,5,5-tetramethyl-1,3,2-dioxaborolan-2-yl, cod = 1,5-cyclooctadiene, dtbpy = 4,4′-di-*t*-butyl-2,2′-bipyridyl.

Two hexyl groups at the 1,9-positions were installed in the design of a fulminene panel to facilitate solubility and site-selective CH-borylation^17^, and we approached the synthesis of the outer cylindrical molecules via the synthetic route shown in Fig. 3. Thus, a phosphonium salt (**1**) and 2-hexylbenzaldehyde were coupled via the Wittig reaction, and subsequent photocyclization afforded 1,9-dihexylfulminene (**2**) in a 45% yield^18^. Boryl groups were installed in a selective manner at the 3,11-positions via Ir-catalysed CH borylation of a diborylated precursor (**3**)^17,19,20^. The boryl handles of precursor **3** were used for macrocyclization via Pt-mediated tetramerization^10,21^, and subsequent reductive eliminations gave [4]CF in a 19% yield. The tetrameric macrocyclic structure of [4]CF was first confirmed by its MALDI MS spectrum, which showed an *m*/*z* value of 1978 (Fig. 3). Although the tetrameric phenacene macrocycles could potentially afford six stereoisomers comprising four diastereomers and two enantiomer pairs (Supplementary Fig. 2)^11^, the simple ^1^H NMR spectrum of [4]CF with six aromatic resonances (four doublets and two singlets) showed the presence of a single diastereomer. Among the four possible structures of [4]CF, isomers with *D*_4_ or *D*_2d_ symmetry can afford this simple spectrum. Upon further chromatographic separations of [4]CF with amylose-loaded silica gels (CHIRALPAK-IA columns), we detected an enantiomer pair, which confirmed the selective production of *D*_4_-symmetric [4]CF. Two enantiomers, **(*P*)-(20,4)** and **(*M*)-(20,4)**, with helical carbon networks were finally separated by preparative HPLC with CHIRALPAK-IA columns (Supplementary Figs. 3 and 4).

### Stereoselective spontaneous assembly of the i-DWNT complex

**Fig. 4:**
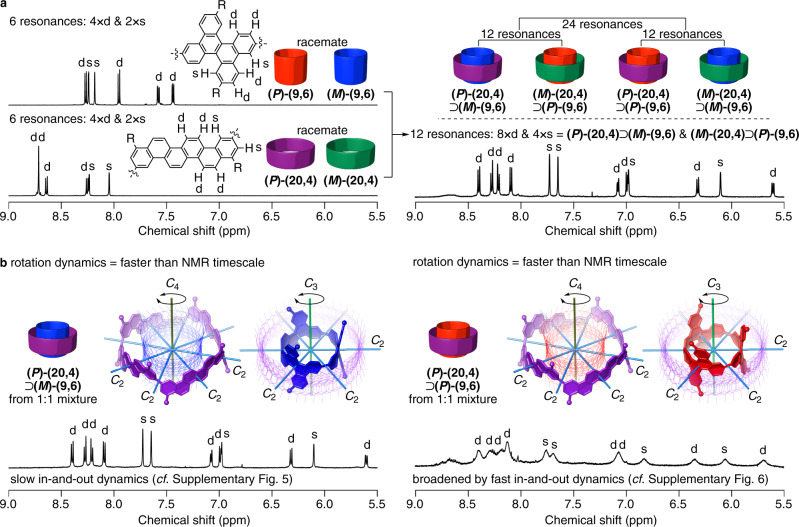
Stereoselective spontaneous assembly of i-DWNT complexes. Spectra were recorded in CD_2_Cl_2_ at 25 °C. **a** ^1^H NMR spectra that demonstrates stereoselective assembly of a single set of i-DWNT diastereomers. Two sets of **(9,6)** and **(20,4)** racemates gave rise to the spectra of six aromatic resonances, and a 1:1 mixture of these two racemates resulted in 12 aromatic resonances. Minor ^4^*J* couplings (<2 Hz) were found with some resonances, which were not included in designations of resonance shapes for clarity. **b** Two diastereomeric complexes were separately prepared to afford two different ^1^H NMR spectra. The spectrum of heterohelical **(*P*)-(20,4)**⊃**(*M*)-(9,6)** was identical to that of the 1:1 mixture of racemates, and the spectrum of homohelical **(*P*)-(20,4)**⊃**(*P*)-(9,6)** confirmed the absence of these resonances in the spectrum of the racemate mixture. See also Supplementary Figs. 5 and 6.

We thus obtained four helical CNT molecules: a pair of enantiomers of small-bore [3]C^db^C molecules^12^, **(*P*)-** and **(*M*)-(9,6)**, and a pair of enantiomers of large-bore [4]CF molecules, **(*P*)-** and **(*M*)-(20,4)** (see Fig. 2). Upon mixing these four cylindrical molecules in dichloromethane, the i-DWNT complexes were spontaneously assembled unexpectedly in a stereoselective manner. As shown in Fig. 4a, each cylindrical molecule shows six aromatic resonances as a racemate. Heterohelical combinations of **(*P*)-(20,4)**⊃**(*M*)-(9,6)** and **(*M*)-(20,4)**⊃**(*P*)-(9,6)** are diastereoisomers of homohelical combinations of **(*P*)-(20,4)**⊃**(*P*)-(9,6)** and **(*M*)-(20,4)**⊃**(*M*)-(9,6)** (*cf*. Fig. 1), and these four stereoisomers, including two sets of diasteromeric complexes, should afford 24 aromatic resonances in the simplest case. However, when we mixed an equimolar amount of the [3]C^db^C and [4]CF racemates containing four cylindrical molecules, we obtained a ^1^H NMR spectrum comprising 12 aromatic resonances (Fig. 4). This spectrum suggested the presence of specific processes during the complexation, and we sought the origin and the structural identity by separately preparing two diastereomeric complexes, i.e., heterohelical and homohelical i-DWNT complexes. We first mixed heterohelical molecules of **(*P*)-(20,4)** and **(*M*)-(9,6)** and obtained a ^1^H NMR spectrum comprising 12 aromatic resonances (8 × d and 4 × s) that perfectly matched the one observed from the racemate mixture. This observation thus showed that the complex formed in the racemate mixture was an enantiomeric mixture of **(*P*)-(20,4)**⊃**(*M*)-(9,6)** and **(*M*)-(20,4)**⊃**(*P*)-(9,6)**. We next mixed homohelical molecules of **(*P*)-(20,4)** and **(*P*)-(9,6)** and indeed obtained a different ^1^H NMR spectrum. The spectrum was composed of 12 broad aromatic resonances appearing at different chemical shifts. Therefore, the spectrum confirmed that homohelical complexes, i.e., **(*P*)-(20,4)**⊃**(*P*)-(9,6)** and **(*M*)-(20,4)**⊃**(*M*)-(9,6)**, were not observed in the racemate mixture. In a separate set of NMR experiments, we further confirmed two facts: (1) when present, unbound free forms of **(*P*)-(20,4)** and **(*M*)-(9,6)** should be observed separately from its complexed form of **(*P*)-(20,4)**⊃**(*M*)-(9,6)** (Supplementary Fig. 5) and (2) the broadened resonances of **(*P*)-(20,4)**⊃**(*P*)-(9,6)** became sharpened upon cooling, which showed that the broadening was due to rapid in-and-out exchange with unbound free forms (Supplementary Fig. 6). These additional observations thus completed the spectral identifications of heterohelical and homohelical complexes and, more importantly, stereoselective formation of heterohelical (*P*)/(*M*)- and (*M*)/(*P*)-i-DWNT.

Observations of 12 aromatic resonances with i-DWNT complexes also showed the presence of concentric rotational dynamics^22–25^. Thus, with the given point symmetry of the cylindrical molecules [**(20,4)** = *D*_4_ and **(9,6)** = *D*_3_], we should expect 84 aromatic resonances for the static, *C*_1_ symmetric state. The observations of 12 aromatic resonances with heterohelical **(*P*)-(20,4)**⊃**(*M*)-(9,6)** show that the *C*_4_ and *C*_3_ axes are present for **(*P*)-(20,4)** and **(*M*)-(9,6)**, respectively, which shows that the relative rotational motions of two cylindrical molecules take place at a faster speed than the NMR timescale (see also Supplementary Fig. 5)^26^. The rotational dynamics of homohelical **(*P*)-(20,4)**⊃**(*P*)-(9,6)** were likewise concluded by the simple ^1^H NMR spectrum with 12 aromatic resonances (see also Supplementary Fig. 6).

### Energetics of spontaneous assembly of the i-DWNT complex

**Fig. 5:**
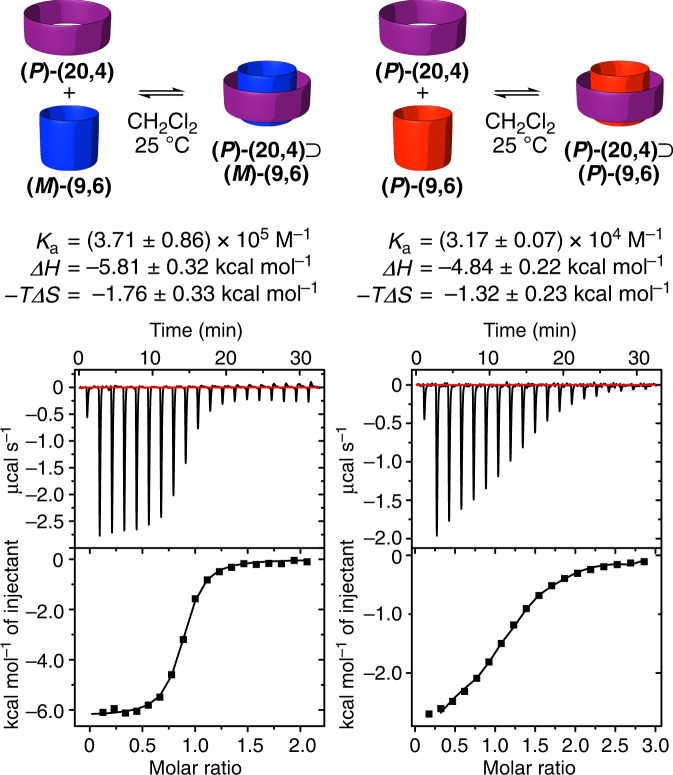
Spontaneous assembly of i-DWNT complexes. Thermodynamic parameters and titration data of ITC analyses are shown.

The thermodynamic origins of stereoselective i-DWNT complexation were revealed by isothermal titration calorimetry (ITC) analyses^27,28^. The association constant (*K*_a_) and the thermodynamic parameters (*ΔH* and −*TΔS*) for the association were derived for both complexes by triplicate titration experiments with ITC. For the heterohelical combination of **(*P*)-(20,4)**⊃**(*M*)-(9,6)**, the values were *K*_a_ = 4 × 10^5^ M^−1^, *ΔH* = −6 kcal mol^−1^ and −*TΔS* = −2 kcal mol^−1^ (Fig. 5), and for the homohelical combination of **(*P*)-(20,4)**⊃**(*P*)-(9,6)**, the values were *K*_a_ = 3 × 10^4^ M^−1^, *ΔH* = −5 kcal mol^−1^ and −*TΔS* = −1 kcal mol^−1^ (Fig. 5). The association constant of the heteroherical i-DWNT complex was thus one order of magnitude higher than that of the homohelical i-DWNT complex, which explained well the absence of **(*P*)-(20,4)**⊃**(*P*)-(9,6)** in the ^1^H NMR spectrum of the 1:1 mixture of the racemates (Fig. 4a). The ITC result was also important to clarify that the enthalpic difference (*ΔΔH*) of incommensurate carbon bilayers with diastereomeric orientations can be as large as 1 kcal mol^−1^ even for short, finite cylindrical molecules. In previous studies of infinite i-DWNTs with transmission electron microscopy, tomographic analyses led to the proposal that homohelical CNT combinations were energetically preferred over heterohelical combinations^29,30^. This previous proposal contradicts the present quantitative thermodynamic data of i-DWNT segments, and two explanations might be possible: (1) the stereoselectivity of i-DWNT formation may be kinetically determined, which may prefer homohelical combinations, and (2) the proposal made for the 16 i-DWNT specimens was not statistically sufficient. However, infinite DWNTs are usually produced at high temperatures (~1000 °C)^31^, and the stereoselective kinetic formation of one diastereomeric form under such severe conditions is not readily expected for the production of i-DWNTs differing only in carbon arrangements such as (*P*)/(*M*) *vs*. (*P*)/(*P*). In the future, diastereoselectivity in the incommensurate pairs of carbon bilayers should be carefully analysed.

### Crystal structures of the i-DWNT complex

**Fig. 6:**
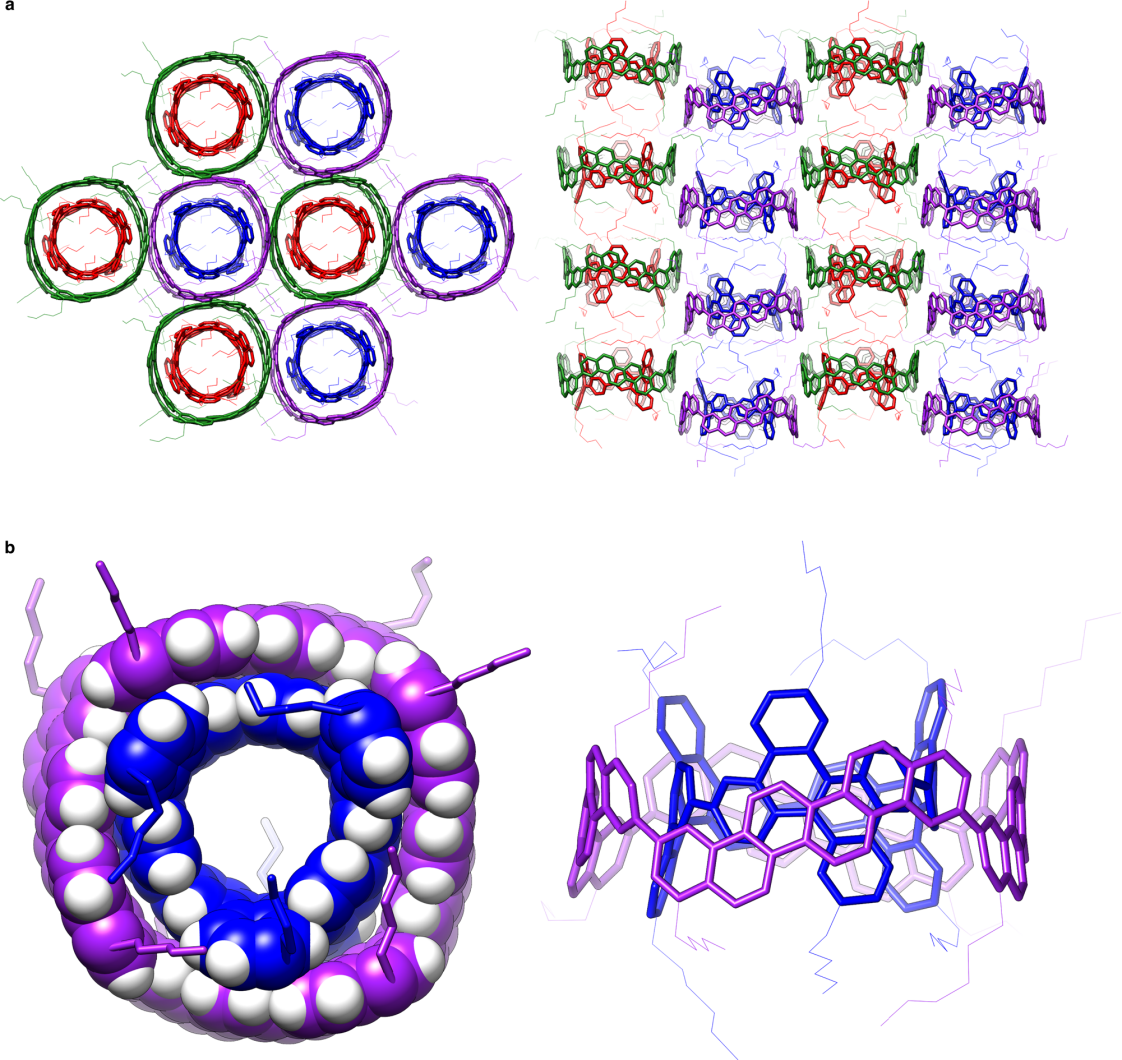
Crystal structures of i-DWNT complexes. **a** Upon mixing four cylindrical molecules, racemates of **(*P*)-(20,4)**⊃**(*M*)-(9,6)** and **(*M*)-(20,4)**⊃**(*P*)-(9,6)** were selectively formed and assembled in a pairwise manner to fill the crystal space of monoclinic *P*2_1_/*n*. **b** Top view and side view of the molecular structure of **(*P*)-(20,4)**⊃**(*M*)-(9,6)**.

The molecular structures of the i-DWNT complexes were fully revealed by X-ray crystallographic analyses. Taking into account the stereoselective assembly of heterohelical combinations (vide supra) and the effective crystal growth of racemates^12,32–34^, we mixed an equimolar amount of four molecules, i.e., **(*P*)-(20,4)**, **(*M*)-(20,4)**, **(*P*)-(9,6)** and **(*M*)-(9,6)**, in chloroform, and a single crystal was grown at 25 °C with vapour diffusion of acetonitrile. As expected, the heterohelical i-DWNT complexes of **(*P*)-(20,4)**⊃**(*M*)-(9,6)** and **(*M*)-(20,4)**⊃**(*P*)-(9,6)** were assembled in a stereoselective manner, and a pair of enantiomers effectively filled the crystal space by forming a glide plane with their pairwise locations (Fig. 6a)^35^. Enantiomers were also segregated into homochiral columns that appeared alternately in the crystals. Most importantly, the crystal structure revealed the molecular structure of the i-DWNT segments having ideal coaxial structures. The tight vdW contacts between the carbon layers possessed an ideal interlayer distance (Supplementary Fig. 7), which explained the large association constant of 10^5^ M^−1^ in solution. In the crystal, the i-DWNT complex was observed as a static structure with *C*_1_ point symmetry, which, in turn, also confirmed the dynamic relative rotations of assembled cylinders in solution to regenerate the *D*_4_/*D*_3_ symmetry of each component.

## Discussion

In summary, we report a molecular version of i-DWNT composed of incommensurate carbon bilayers. A helical CNT segment was designed by adopting fulminene ([6]phenacene) as contorted panels, and the cylindrical molecule ([4]CF) was synthesised to realise a large-bore, ideal diameter to encapsulate a small-bore helical CNT segment ([3]C^db^C). Coaxial assembly of i-DWNT enantiomers spontaneously proceeded upon mixing four CNT molecules in solution, and the assembly favoured a single set of diastereomers via discrimination of helical carbon arrangements in the pairing incommensurate layers. Consequently, heterohelical combinations of (*P*)/(*M*) and (*M*)/(*P*) were preferred over homohelical combinations of (*P*)/(*P*) and (*M*)/(*M*), and an enthalpic origin for the discrimination was quantitatively disclosed. This result may prompt reconsiderations of previous proposals of homohelical preferences for infinite i-DWNTs^29–31^. Spectral changes were noted upon formation of the i-DWNT complex (Supplementary Fig. 13), and in-depth investigations of origins may shed light on electronic interactions between carbon bilayers in future. The single crystal of i-DWNT complexes was grown from a mixture of four CNT molecules and unequivocally revealed the coaxial assembled structures of heterohelical i-DWNT composed of an incommensurate carbon bilayer. Structural information with atomic precision along with quantitative measures of association thermodynamics have deepened our understanding of incommensurate nanocarbon assembly^1–3^. Further studies of incommensurate carbon bilayers and the unique rotational dynamics of i-DWNTs should be explored by designing nanocarbon molecules with discrete structures with atomic precision^36–39^.

## Methods

### Materials

Isomers of [3]C^db^C [**(*P*)-(9,6)** and **(*M*)-(9,6)**] were synthesised and isolated according to a previously reported procedure^12^.

### Synthesis of [4]CF

A mixture of diborylfulminmene **3** (1.00 g, 1.34 mmol), PtCl_2_(cod) (501 mg, 1.34 mmol) and CsF (1.22 g, 8.03 mmol) in THF (67 mL) was refluxed for 24 h. After the addition of water (*ca*. 30 mL) at ambient temperature, the resulting precipitates were collected by filtration and washed with water (*ca*. 100 mL) and methanol (*ca*. 100 mL). The precipitates were then stirred in *o*-dichlorobenzene (110 mL) at 100 °C for 3 h. Triphenylphosphine (3.50 g, 13.4 mmol) was added to the mixture at ambient temperature, and the mixture was stirred at ambient temperature for 30 min and at 100 °C for 24 h. Methanol (*ca*. 500 mL) was added to the mixture at ambient temperature, and the resulting precipitates were collected by filtration. The crude material was purified by silica gel column chromatography (eluent: 20% chloroform/hexane) and gel permeation chromatography to afford [4]CF as a racemate of **(*P*)-(20,4)** and **(*M*)-(20,4)** (129 mg, 65.2 μmol, 19%). After further purification of enantiomers by preparative HPLC (Supplementary Fig. 3), the absolute configurations of **(*P*)-(20,4)** and **(*M*)-(20,4)** were deduced by CD spectra with the aid of time-dependent density functional calculations (Supplementary Fig. 4). Physical data of [4]CF: ^1^H NMR (600 MHz, CDCl_3_, 25 °C) δ 8.69 (d, *J* = 9.7 Hz, 8H), 8.66 (d, *J* = 9.7 Hz, 8H) 8.61 (d, *J* = 9.6 Hz, 8H), 8.22 (d, *J* = 9.6 Hz, 8H), 8.19 (s, 8H), 7.99 (s, 8H), 3.43–3.35 (m, 8H), 3.31–3.25 (m, 8H), 1.98–1.91 (m, 16H), 1.62–1.55 (m, 16H), 1.38–1.47 (m, 32H), 0.95 (t, *J* = 7.2 Hz, 24H); ^13^C NMR (151 MHz, CDCl_3_, 25 °C) δ 140.57, 140.41, 131.60, 129.53, 129.03, 128.95, 128.17, 125.54 (CH), 124.55 (CH), 123.27 (CH), 122.39 (CH), 122.20 (CH), 121.16 (CH), 33.80 (CH_2_), 32.01 (CH_2_), 31.46 (CH_2_), 29.78 (CH_2_), 22.97 (CH_2_), 14.35 (CH_3_); IR (neat): 2923, 2359, 1407, 807 cm^−1^; HRMS (MALDI-TOF) (*m/z*): [M]^+^ calcd for C_152_H_152_ 1977.1889, found 1977.1931. NMR spectra are shown in Supplementary Figs. 11 and 12.

### NMR analysis of i-DWNT complexes

^1^H NMR spectra of i-DWNT complexes were recorded on a JEOL RESONANCE JNM-ECA 600 II equipped with an UltraCOOL probe. The spectrum of an equimolar mixture of **(*P*)-(20,4)**, **(*M*)-(20,4)**, **(*P*)-(9,6)** and **(*M*)-(9,6)** was recorded at a total concentration of 1 mM in CD_2_Cl_2_ (Fig. 4a). Spectra of **(*P*)-(20,4)**⊃**(*M*)-(9,6)** and **(*P*)-(20,4)**⊃**(*P*)-(9,6)** were also recorded at a total concentration of 1 mM in CD_2_Cl_2_ (Fig. 4b).

### Isothermal titration calorimetry

The association thermodynamics of **(*P*)-(20,4)**⊃**(*M*)-(9,6)** and **(*P*)-(20,4)**⊃**(*P*)-(9,6)** in CH_2_Cl_2_ were analysed by ITC performed on a Malvern MicroCal iTC200 microcalorimeter. For **(*P*)-(20,4)**⊃**(*M*)-(9,6)**, to a solution of **(*P*)-(20,4)** (0.208 mM) in a cell of the microcalorimeter, we added a solution of **(*M*)-(9,6)** (2.26 mM) to record the titration curves. For **(*P*)-(20,4)**⊃**(*P*)-(9,6)**, 0.256 mM of **(*P*)-(20,4)** and 3.65 mM of **(*P*)-(9,6)** were used. Sigmoidal titration curves were fitted by ORIGIN software to afford the association constant, *K*_a_, and the thermodynamic parameters, *ΔH* and *ΔS*. The titration experiments were performed three times for each specimen, and averaged values with the standard deviation were obtained. Representative titration curves and thermodynamic parameters are shown in Fig. 5. The association constants at 10^5^ M^−1^ were one to four orders of magnitude larger than those recorded with commensurate pairs of [*n*]cycloparaphenylenes^16^.

### Crystallographic analyses

Single crystals of a racemate of **(*P*)-(20,4)**⊃**(*M*)-(9,6)** and **(*M*)-(20,4)**⊃**(*P*)-(9,6)** that were suitable for X-ray analysis were grown from a solution of a 1:1:1:1 mixture of **(*P*)-(20,4)**, **(*M*)-(20,4)**, **(*P*)-(9,6)** and **(*M*)-(9,6)** in chloroform (*ca*. 1 mg/mL) via gradual introduction of acetonitrile by vapour diffusion at 25 °C. The single crystal was mounted on a thin polymer tip with cryoprotectant oil and frozen via flash cooling. Diffraction analyses were conducted with synchrotron X-ray sources (BL26B1, SPring-8) at 100 K by using a diffractometer equipped with a Dectris EIGER X 4 M PAD detector. The diffraction data were processed with the XDS software program^40^. The structure was solved by direct methods with SHELXT^41^ and refined by full-matrix least squares on *F*^2^ using the SHELXL-2014/7 programme suite^42^ running with Yadokari-XG 2009^43^. In the refinements, disordered alkyl chains and solvent molecules were partly restrained by SIMU, DFIX and DANG. The non-hydrogen atoms were analysed anisotropically, and hydrogen atoms were located at the calculated positions and refined with a riding model. The crystal structure is shown in Fig. 6 and Supplementary Fig. 7. Crystal data and structure refinement are listed in Supplementary Table 1.

## Supplementary information

Supplementary Information

## Data Availability

Supplementary methods, spectra and computational data are provided in the Supplementary Information. Crystallographic data of a racemate of **(*****P*****)-(20,4)**⊃**(*****M*****)-(9,6)** and **(*****M*****)-(20,4)**⊃**(*****P*****)-(9,6)** have been deposited at the Cambridge Crystallographic Data Centre with deposition number CCDC 2034189. These data can be obtained free of charge from the Cambridge Crystallographic Data Centre via www.ccdc.cam.ac.uk/structures/.

## References

[1] Geim AK, Grigorieva IV (2013). Van der Waals heterostructures. Nature.

[2] Kim C-J (2016). Chiral atomically thin films. Nat. Nanotechnol..

[3] Cao Y (2018). Unconventional superconductivity in magic-angle graphene superlattices. Nature.

[4] Iijima S (1991). Helical microtubules of graphitic carbon. Nature.

[5] Saito R, Dresselhaus G, Dresselhaus MS (1993). Electronic structure of double-layer graphene tubules. J. Appl. Phys..

[6] Shen C, Brozena AH, Wang Y (2011). Double-walled carbon nanotubes: challenges and opportunities. Nanoscale.

[7] Komatsu N (2010). Stereochemistry of carbon nanotubes. Jpn. J. Appl. Phys..

[8] Koshino M, Moon P, Son Y-W (2015). Incommensurate double-wall carbon nanotubes as one-dimensional moiré crystals. Phys. Rev. B.

[9] Liu K (2014). Van der Waals-coupled electronic states in incommensurate double-walled carbon nanotubes. Nat. Phys..

[10] Hitosugi S, Nakanishi W, Yamasaki T, Isobe H (2011). Bottom-up synthesis of finite models of helical (*n*,*m*)-single-wall carbon nanotubes. Nat. Commun..

[11] Sun Z, Matsuno T, Isobe H (2018). Stereoisomerism and structures of rigid cylindrical cycloarylenes. Bull. Chem. Soc. Jpn..

[12] Kogashi K, Matsuno T, Sato S, Isobe H (2019). Narrowing segments of helical carbon nanotubes with curved aromatic panels. Angew. Chem. Int. Ed..

[13] Saito, R. Dresselhaus, G., & Dresselhaus, M. S. *Physical Properties of Carbon Nanotubes* (Imperial College Press, 1998).

[14] Matsuno T (2014). Geometric measures of finite carbon nanotube molecules: a proposal for length index and filling indexes. Pure Appl. Chem..

[15] Hirahara K (2006). Chirality correlation in double-wall carbon nanotubes as studied by electron diffraction. Phys. Rev. B.

[16] Hashimoto S, Iwamoto T, Kurachi D, Kayahara E, Yamago S (2017). Shortest double-walled carbon nanotubes composed of cycloparaphenylenes. ChemPlusChem.

[17] Ishiyama T (2002). Mild iridium-catalyzed borylation of arenes. High turnover numbers, room temperature reactions, and isolation of a potential intermediate. J. Am. Chem. Soc..

[18] Mallory, F. B. & Mallory, C. W. Photocyclization of stilbenes and related molecules in *Organic Reactions*, Vol. 30, pp. 1–456 (Wiley, 1984).

[19] Matsuno T, Kamata S, Hitosugi S, Isobe H (2013). Bottom-up synthesis and structures of π-lengthened tubular macrocycles. Chem. Sci..

[20] Hitosugi S, Nakamura Y, Matsuno T, Nakanishi W, Isobe H (2012). Iridium-catalyzed direct borylation of phenacenes. Tetrahedron Lett..

[21] Yamago S, Watanabe Y, Iwamotno T (2010). Synthesis of [8]cycloparaphenylene from a square‐shaped tetranuclear platinum complex. Angew. Chem. Int. Ed.

[22] Isobe H, Hitosugi S, Yamasaki T, Iizuka R (2013). Molecular bearings of finite carbon nanotubes and fullerenes in ensemble rolling motion. Chem. Sci..

[23] Matsuno T, Fujita M, Fukunaga K, Sato S, Isobe H (2018). Concyclic CH-π arrays for single-axis rotations of a bowl in a tube. Nat. Commun..

[24] Sun Z (2019). Unbiased rotational motions of an ellipsoidal guest in a tight yet pliable host. Angew. Chem. Int. Ed..

[25] Matsuno T, Nakai Y, Sato S, Maniwa Y, Isobe H (2018). Ratchet-free solid-state inertial rotation of a guest ball in a tight tubular host. Nat. Commun..

[26] Oki, M. *Applications of Dynamic NMR Spectroscopy to Organic Chemistry* (VCH, 1985).

[27] Schmidtchen, F. P. Isothermal titration calorimetry in supramolecular chemistry. in *Supramolecular Chemistry: From Molecules to Nanomaterials* (eds Steed, J. W. & Gale, P. A.) Vol. 1, pp 275–296 (Wiley, 2012).

[28] Matsuno, T., Sato, S. & Isobe, H. Curved π-receptors. in *Comprehensive Supramolecular Chemistry II* (eds Atwood, J. L., Gockel, G. W. & Barbour, L. J.) Vol. 3, pp 311–328 (Elsevier, 2017).

[29] Liu Z (2005). Determination of optical isomers for left-handed or right-handed chiral double-wall carbon nanotubes. Phys. Rev. Lett..

[30] Rochal S (2019). Chirality manifestation in elastic coupling between the layers of double-walled carbon nanotubes. Nanoscale.

[31] Sugai T, Yoshida H, Shimada T, Okazaki T, Shinohara H (2003). New synthesis of high-quality double-walled carbon nanotubes by high-temperature pulsed arc discharge. Nano Lett..

[32] Matthews BW (2009). Racemic crystallography–Easy crystals and easy structures: What’s not to like?. Protein Sci..

[33] Hitosugi S, Yamasaki T, Isobe H (2012). Bottom-up synthesis and thread-in-bead structure of finite (*n*,0)-zigzag single-wall carbon nanotubes. J. Am. Chem. Soc..

[34] Sato S, Yamasaki T, Isobe H (2014). Solid-state structures of peapod bearings composed of finite single-wall carbon nanotube and fullerene molecules. Proc. Natl Acad. Sci. USA.

[35] Aroyo, M. I. International Tables for Crystallography, Volume A: Space Group Symmetry (IUCr, 2016).

[36] Cumings J, Zettl A (2000). Low-friction nanoscale linear bearing realized from multiwall carbon nanotubes. Science.

[37] Fennimore AM (2003). Rotational actuators based on carbon nanotubes. Nature.

[38] Saito R, Matsuo R, Kimura T, Dresselhaus G, Dresselhaus MS (2001). Anomalous potential barrier of double-wall carbon nanotube. Chem. Phys. Lett..

[39] Kolmogorov AN, Crespi VH (2000). Smoothest bearings: Interlayer sliding in multiwalled carbon nanotubes. Phys. Rev. Lett..

[40] Kabsch W (1993). Automatic processing of rotation diffraction data from crystals of initially unknown symmetry and cell constants. J. Appl. Cryst..

[41] Sheldrick GM (2015). SHELXT – Integrated space-group and crystal-structure determination. Acta Cryst..

[42] Sheldrick, G. M. & Schneider, T. R. in *Macromolec. Crystal. B Book Series: Methods in Enzymology*, Vol. 277, pp. 319–343 (Academic Press, 1997). 18488315

[43] Kabuto C, Akine S, Nemoto T, Kwon E (2009). Yadokari-XG, Software for crystal structure analyses. J. Crystallogr. Soc. Jpn.

