# Supplementary material for: Stereoselectivity in spontaneous assembly of rolled incommensurate carbon bilayers
Source: Nat Commun. 2021 Mar 10;12:1575. doi: 10.1038/s41467-021-21889-8 (PMC7946902; doi:10.1038/s41467-021-21889-8)
Supplement: Supplementary file 1 — Supplementary Information [file 41467_2021_21889_MOESM1_ESM.pdf]

Supplementary Information

Stereoselectivity in spontaneous assembly of rolled incommensurate carbon bilayers

Taisuke Matsuno,* Yutaro Ohtomo, Maki Someya, Hiroyuki Isobe*

Table of Contents

Supplementary Methods 1

General 1

Synthesis 2

Theoretical calculations 3

Spectral features of i-DWNT 3

Supplementary Figures 4

Supplementary Tables 18

Supplementary References 36

Supplementary Methods

General

Flash silica gel column chromatography was performed on silica gel 60N (spherical and neutral gel, 40-50 μm, Kanto). Gel permeation chromatography (GPC) was performed on Japan Analytical Industry LC-9104 with JAIGEL 1H, 2H and 2.5 H polystyrene column (eluent: chloroform). High pressure liquid chromatography (HPLC) was performed with chiral columns (CHIRALPAK-IA, Daicel) by using a 4.6 × 250 mm column for analytical scales and a 20 × 250 mm column for preparative scales. The analytical HPLC was performed at 40 °C in a column oven (JASCO CO2060PLUS) under detection of UV-vis absorption (JASCO MD2018PLUS) and circular dichromism (CD) intensities (JASCO CD2095PLUS) with a flow rate of 1.0 mL/min. The preparative HPLC was performed at ambient temperature under detection of UV-vis (JASCO UV2075PLUS) with a flow rate of 10 mL/min. High resolution mass spectra (HRMS) were performed on a Bruker Daltonics autoflex speed using the matrix assisted laser desorption ionization (MALDI) method with pyrene as a matrix under a reflector positive mode or on a Bruker micrOTOF II spectrometer equipped with an APCI probe equipped with a DirectProbe (DIP). UV-vis and CD spectra were recorded on JASCO V-670 and JASCO J-1500 spectropolarimeters, respectively. Nuclear magnetic resonance (NMR) spectra were recorded on JEOL RESONANCE JNM-ECA 600 II equipped with

the UltraCOOL probe (UC5AT). Chemical shift values were given with respect to internal $CHCl_3$ (7.26) and $CHDCl_2$ (5.32) for ^{1}H NMR and $CDCl_3$ (77.16) and for ^{13}C NMR. Methyl (CH_3), methylene (CH_2) and methine (CH) signals in ^{13}C NMR spectra were assigned by DEPT spectra. Infrared (IR) spectroscopy was performed on JASCO FT/IR-4700 equipped with JASCO ATR PRO ONE.

Synthesis

1,9-Dihexylfulminene (**2**)

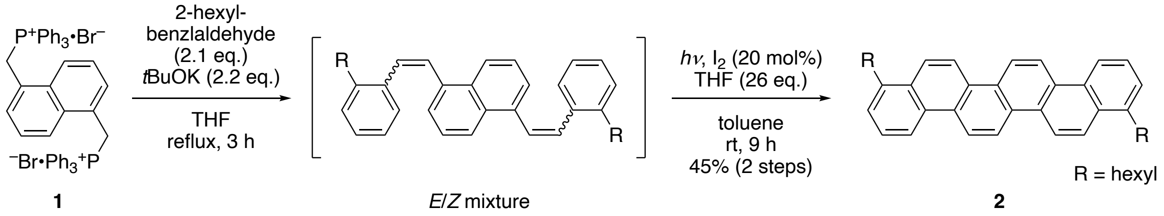

Precursors, *i.e.*, compound **1** and 2-hexylbenzaldehyde, were prepared by methods reported in the literatures[1,2]. To a suspension of **1** (2.01 g, 2.40 mmol) in THF (70 mL) was added *t*-BuOK (1.0 M in THF, 5.3 mL, 5.3 mmol), and the mixture was stirred for 1 h at ambient temperature. A solution of 2-hexylbenzaldehyde (0.967 g, 5.08 mmol) in THF (40 mL) was then added to the mixture, and the mixture was refluxed for 3 h. After addition of 1 M aq. HCl (*ca.* 100 mL), the organic compounds were extracted with ethyl acetate (*ca.* 100 mL × 3). The organic layer was dried over Na_2SO_4 and concentrated in vacuo. The crude material was passed through silica gel pad (eluent: 50% chloroform/hexane) to afford a mixture of *E*/*Z* isomers of a stilbene derivative. The stilbene derivative was subjected to following photocyclization by mixing with iodine (0.120 g, 0.473 mmol) in THF (5.00 mL)/toluene (500 mL) under irradiation of high-pressure Hg-lamp (400 W, SEN Lights Corp.) for 60 h. The solvent was removed by evaporation, and the crude solid material was washed with toluene (*ca.* 100 mL) to afford a fulminene derivative (**2**) as a white solid (0.529 g, 1.06 mmol, 45% yield from **1**). Physical data of **2**: ^{1}H NMR (600 MHz, $CDCl_3$, 25 °C) δ 9.02 (d, J = 9.6 Hz, 2H), 9.00 (d, J = 9.6 Hz, 2H), 8.89 (d, J = 8.9 Hz, 2H), 8.78 (d, J = 7.6 Hz, 2H), 8.32 (d, J = 8.9 Hz, 2H), 7.67 (dd, J = 7.6 Hz, 7.6 Hz, 2H), 7.52 (d, J = 7.6 Hz, 2H), 3.22 (t, J = 7.9 Hz, 4H), 1.82-1.87 (m, 4H), 1.52-1.48 (m, 4H), 1.42-1.33 (m, 8H), 0.92 (t, J = 7.2 Hz, 6H); Due to low solubility of **2**, ^{13}C NMR spectrum was not obtained. IR (neat) 2953, 2926, 2855, 1715, 1278, 1098, 785 cm^{-1}; HRMS (APCI) (*m/z*): $[M+H]^+$ calcd for $C_{38}H_{41}$ 497.3184, found 497.3203.

3,11-Bis(4,4,5,5-tetramethyl-1,3,2-dioxaborolan-2-yl)-1,9-dihexylfulminene (**3**)

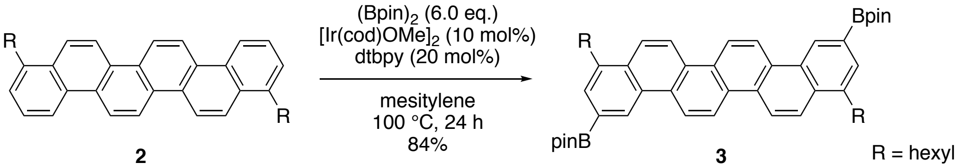

A mixture of **2** (1.91 g, 3.85 mmol), bis(pinacolato)diboron (5.86 g, 23.0 mmol), $[Ir(cod)OMe]_2$ (0.260 mg, 0.392 mmol), dtbpy (0.208 mg, 0.774 mmol) in mesitylene (70 mL) was stirred at 100 °C for 24 h. After addition of methanol (*ca.* 100 mL), the resultant precipitates were collected by filtration and washed with methanol (*ca.* 100 mL) to give a borylated fulminene derivative (**3**) as a white solid (2.43 g, 3.25 mmol, 84%). Physical data of **3**: ^{1}H NMR (600 MHz, $CDCl_3$, 25 °C) δ 9.28 (s, 2H), 9.16 (d, J = 9.6 Hz, 2H), 9.01 (d, J = 9.6 Hz, 2H), 8.93 (d, J = 9.6 Hz, 2H), 8.31 (d, J = 9.6 Hz, 2H), 7.90 (s, 2H), 3.21 (t, J = 8.2 Hz, 4H), 1.88-1.81 (m, 4H), 1.53-1.50 (m, 4H), 1.46 (s, 24H), 1.42-1.33 (m, 8H), 0.93 (t, J = 7.2 Hz, 6H); ^{13}C NMR (151 MHz, $CDCl_3$, 25 °C) δ 139.06, 132.36, 132.10 (CH), 130.28, 129.47, 129.28 (CH), 128.88, 128.09, 123.54 (CH), 122.68, 122.67, 122.15 (CH), 84.15, 33.82 (CH_2), 31.96 (CH_2), 31.75 (CH_2), 29.89 (CH_2), 25.15 (CH_3), 22.85 (CH_2), 14.30 (CH_3); IR (neat) 2921, 2851, 1611, 1338, 1139, 668 cm^{-1}; HRMS (APCI) (m/z): $[M+H]^+$ calcd. for $C_{50}H_{63}B_2O_4$ 749.4914, found 749.4923.

Theoretical calculations

The Gaussian 16 program suite[3] was used for DFT calculations of diastereomers of [4]CF with methyl-substituted models at the B3LYP/6-31G(d,p) level of theory[4,5,6,7,8,9,10,11]. Optimized geometries are shown in Supplementary Fig. 2. Cartesian coordinates of optimized geometries are summarized in Supplementary Tables 2-5.

Spectral features of i-DWNT

UV-vis and CD spectroscopy of i-DWNT was investigated. A mixture of **(*P*)-(20,4)** and **(*M*)-(9,6)** was prepared in dichloromethane by setting concentrations of each component at 1.84×10^{-6} M, and UV-vis and CD spectra were respectively obtained (**spectrum A** in Supplementary Fig. 13). Considering the association constant for i-DWNT **(*P*)-(20,4)⊃(*M*)-(9,6)** ($K_a = 3.71 \times 10^5$ M^{-1}), we can estimate the population of the i-DWNT complex as 32% in **spectrum A**. Separately for reference spectra of free-form components, a solution of **(*P*)-(20,4)** as well as **(*M*)-(9,6)** was also prepared, respectively, in dichloromethane at an identical concentration of 1.84×10^{-6} M. The single-component spectrum of **(*P*)-(20,4)** was added with that of **(*M*)-(9,6)** to afford a summed spectrum of each component (**spectrum B** in Supplementary Fig. 13). Two spectra, **spectrum A** and **spectrum B**, were different: hypochromic effects and subtle red shifts were noted with **spectrum A** in the UV-

vis spectra, and with **spectrum A** in the CD spectra were also noted red shifts. Spectral changes upon i-DWNT complexation suggested the presence of electronic interactions between carbon bilayers. Theoretical calculations of i-DWNT complexation further confirmed the presence of electronic interactions, which further played a key role to determine the stereoselectivity of incommensurate pairs (Supplementary Fig. 14). Because of the rolling dynamics of the i-DWNT complex (Fig. 4b), further theoretical investigations are necessary to fully clarify the i-DWNT structures.

Supplementary Figures

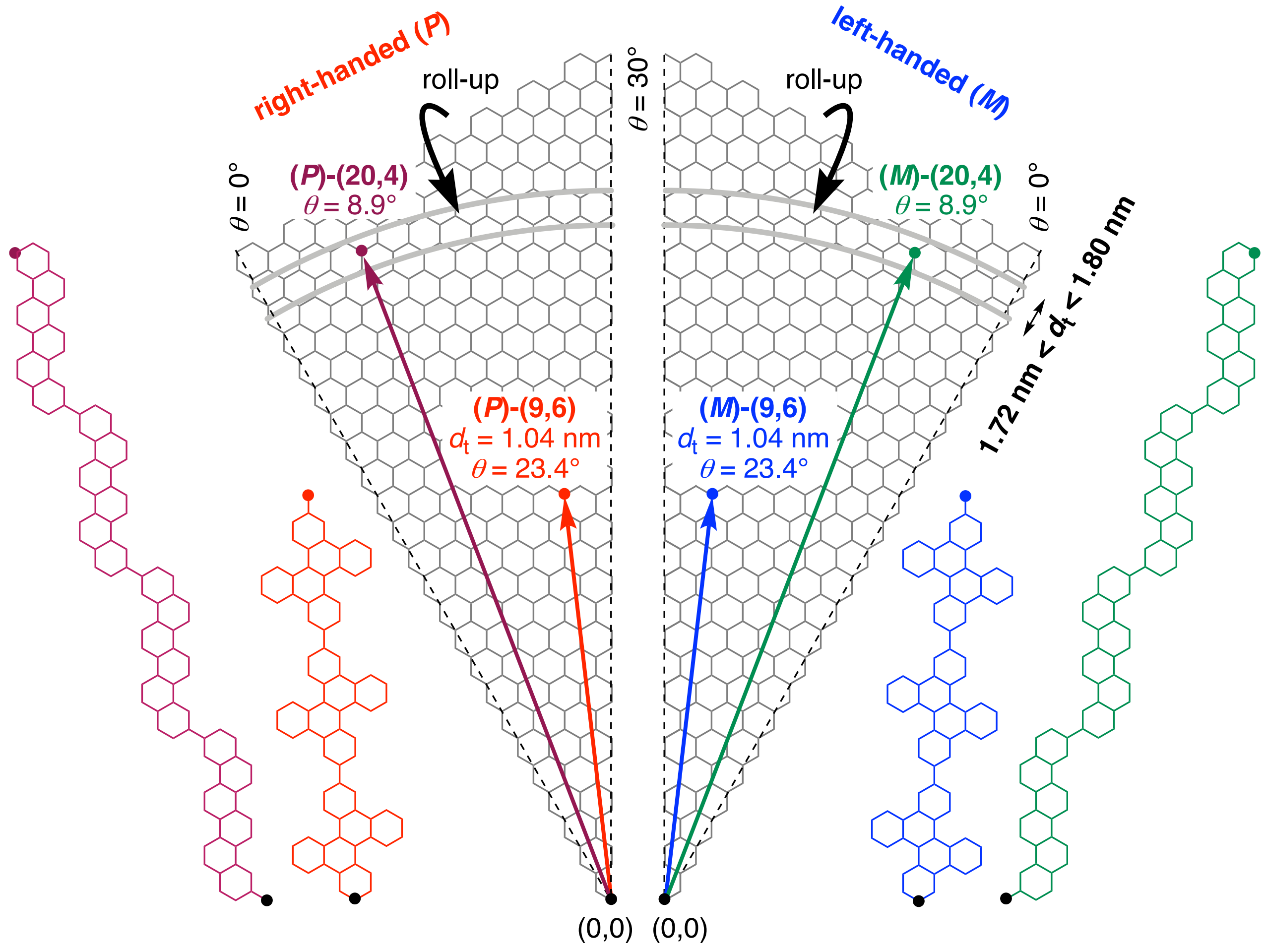

diameter

$$d_t = \frac{a\sqrt{n^2 + m^2 + nm}}{\pi}$$

chiral angle

$$\theta = \tan^{-1}\left(\frac{\sqrt{3}\,m}{m + 2n}\right)$$

Supplementary Fig. 1 | Molecular design by 2D-mapping method. Based on the geometrical diameter of 1.04 for [3]C^{db}C, encapsulating cylindrical structures with diameters of 1.72-1.80 nm were searched by mapping [*n*]phenacene panels on the developed figure. This method allowed us to set [4]CF as an ideal target. For this nomenclature, a relationship of $n > m$ holds, which allows for discriminations of chirality with (*P*)/(*M*) helicity. See also ref. 7 and 11.

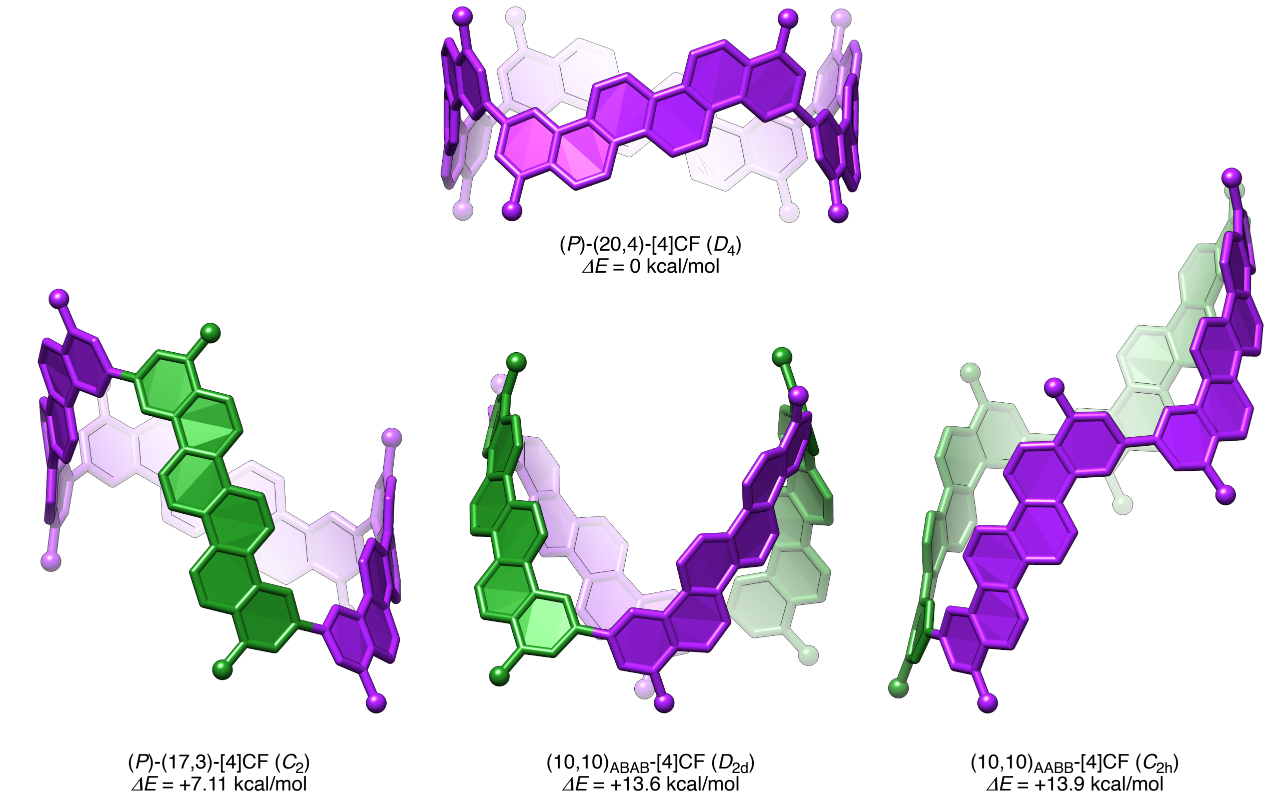

Supplementary Fig. 2 | Structures of four possible diastereomers of [4]CF. Depending on relative orientations of four panels, there emerges four diastereomeric structures for cylindrical [4]CF molecules. Chiral indices are (20,4), (17,3) and (10,10). For helical CNT segments with (20,4) and (17,3), there also emerges enantiomers, and for armchair CNT segments of (10,10), there are two different sets of panel orientations with ABAB and AABB geometries. By adopting methyl groups as model substituents, theoretical structures of [4]CF were obtained at the B3LYP/6-31G(d,p) level of theory. The relative energies exceeding 7 kcal mol^{-1} explain the selective productions of (20,4) enantiomers [***(P)*-(20,4)** and ***(M)*-(20,4)**] through the macrocyclization reaction.

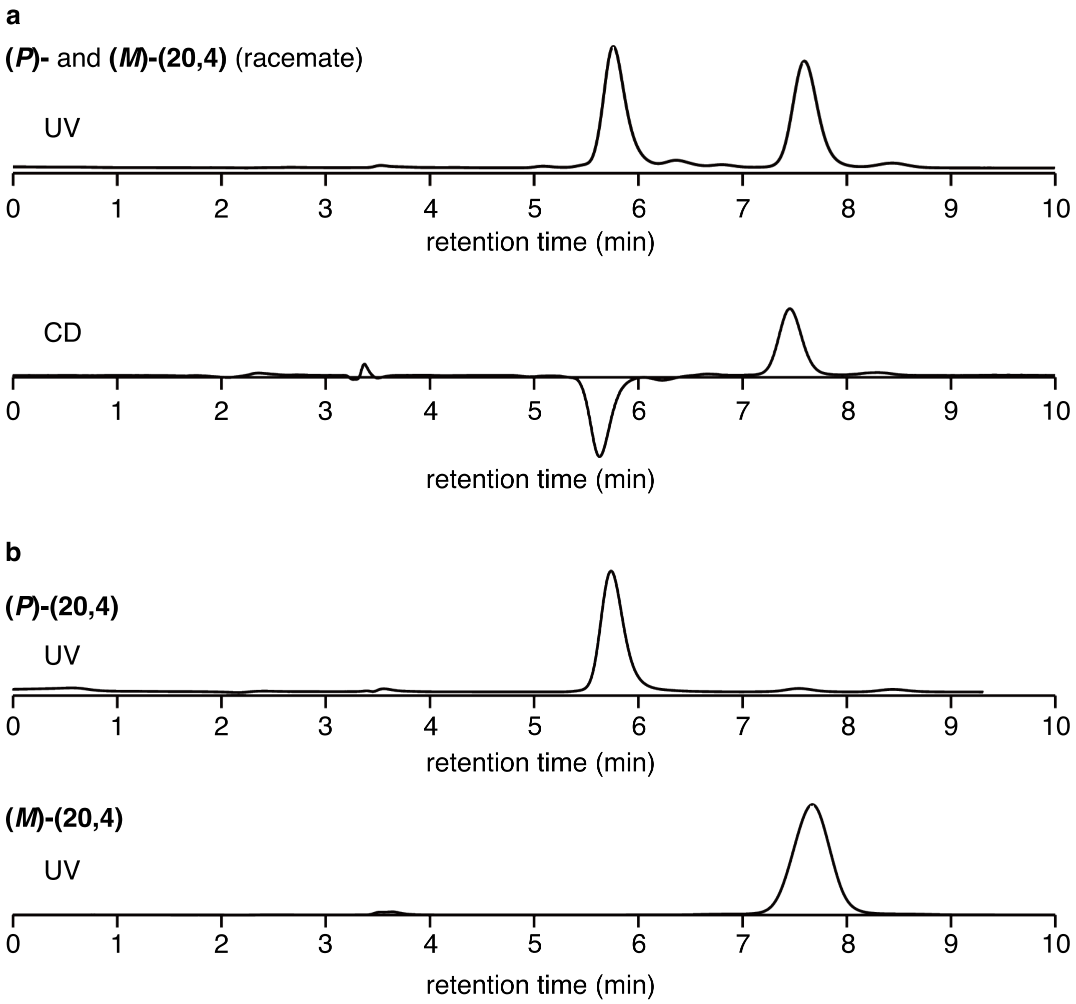

Supplementary Fig. 3 | Chromatograms of [4]CF isomers. a, A chromatogram of a 1:1 mixture of **(*P*)-(20,4)** and **(*M*)-(20,4)** (racemate) after silica gel/GPC purifications. **b,** Chromatograms of the separated enantiomers, **(*P*)-(20,4)** and **(*M*)-(20,4)**, after HPLC purifications.

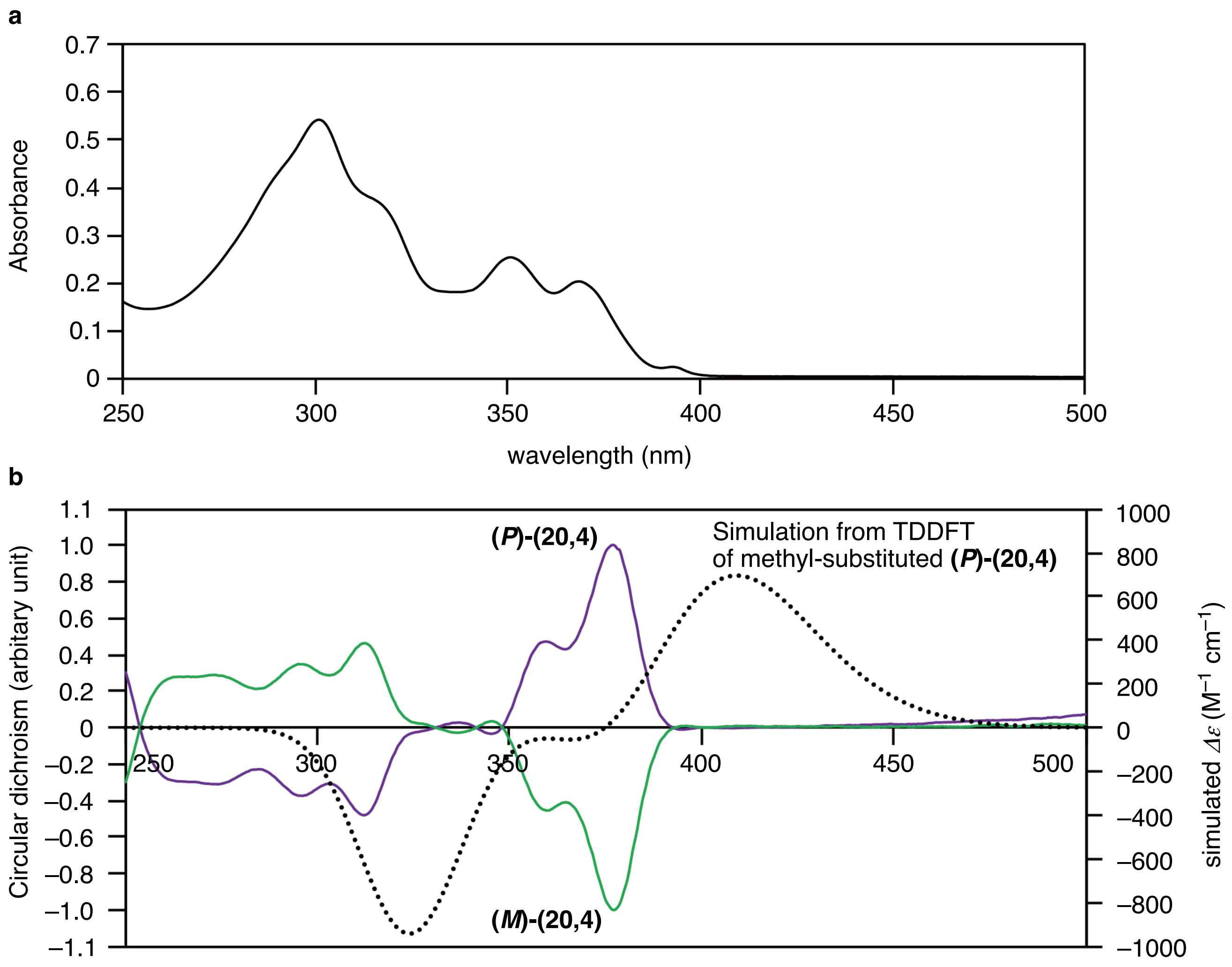

Supplementary Fig. 4 | UV-vis and CD spectra of [4]CF. a, UV-vis spectrum of ***(P)*-(20,4)** in $CHCl_3$ at 25 °C. **b,** CD spectra of ***(P)*-(20,4)** and ***(M)*-(20,4)** in $CHCl_3$ at 25 °C. A simulated spectrum from TD-DFT calculations of ***(P)*-(20,4)** with methyl substituents is shown with a dotted line. Although the theoretical spectrum is expectedly red-shifted due to underestimation of transition energies[12,13,14,15], we assigned the spectrum with a positive CD signal at the lowest transition as the (*P*)-isomer.

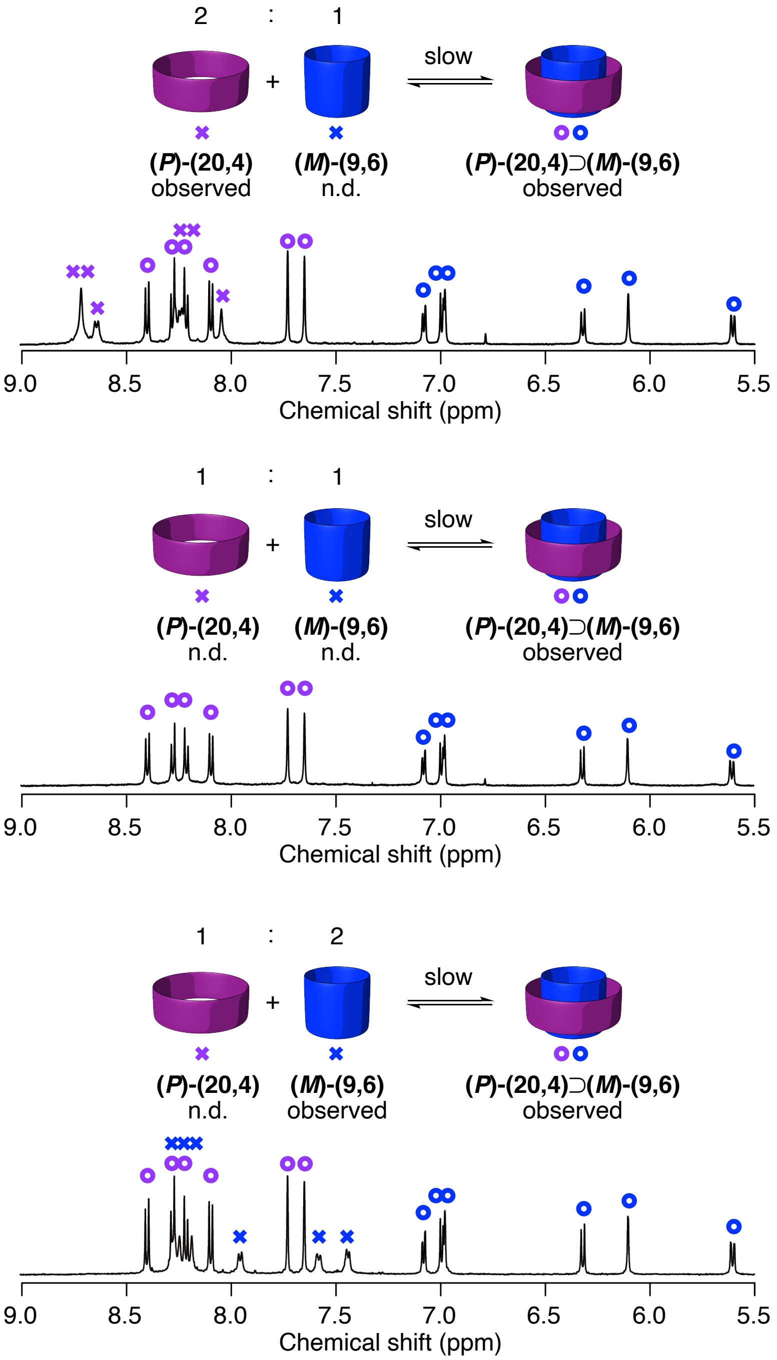

Supplementary Fig. 5 | ^{1}H NMR spectra demonstrating slow in-and-out exchanges with (*P*)-(20,4)⊃(*M*)-(9,6). A reference spectrum of **(*P*)-(20,4)⊃(*M*)-(9,6)** is shown at the middle. When an excess amount of outer **(*P*)-(20,4)** was introduced at 2:1 ratio (top), resonances from free-form **(*P*)-(20,4)** were observed separately from **(*P*)-(20,4)⊃(*M*)-(9,6)**. Likewise, when an excess amount of inner **(*M*)-(20,4)** was introduced at 1:2 ratio (bottom), resonances from free-form **(*P*)-(20,4)** were observed separately from **(*P*)-(20,4)⊃(*M*)-(9,6)**. Spectra were recorded in CD_2Cl_2 at 25 °C with total concentrations of **(*P*)-(20,4)** and **(*M*)-(9,6)** set at 1 mM. Resonance assignments of each component in i-DWNT complexes can be performed by comparison of integral values: **(20,4)** comprises four panels, and **(9,6)** comprises three panels.

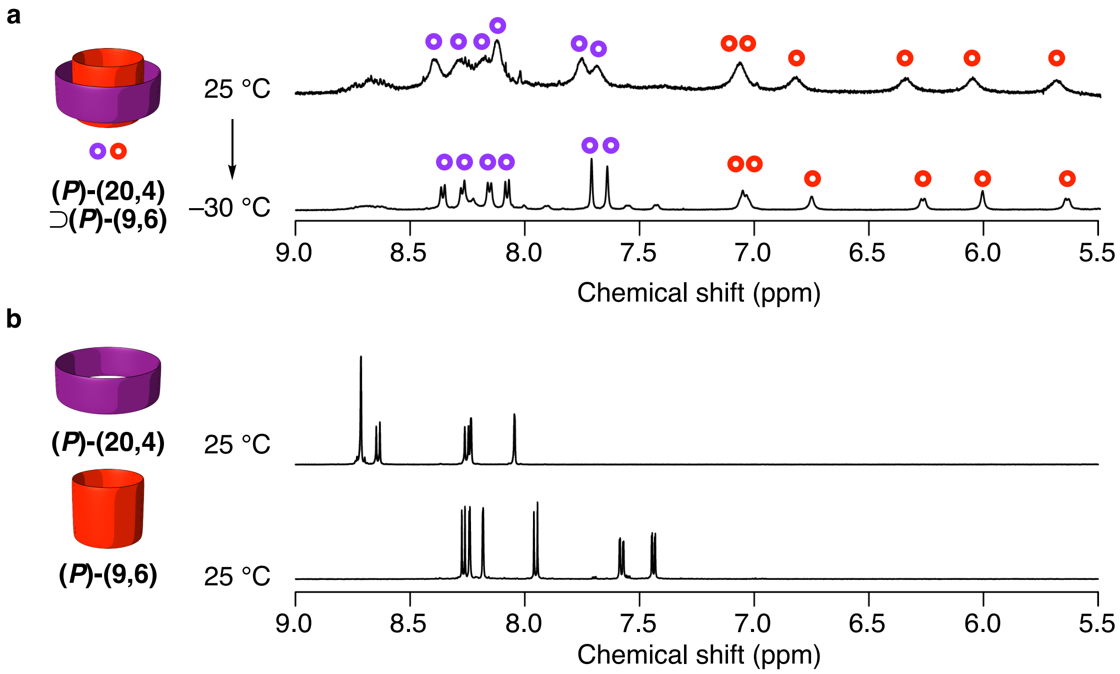

Supplementary Fig. 6 | ^{1}H NMR spectra revealing the origin of resonance broadenings with (*P*)-(20,4)⊃(*P*)-(9,6). a, ^{1}H NMR spectrum of **(*P*)-(20,4)⊃(*P*)-(9,6)** at 25 °C showed broadened resonances. Upon lowering the temperature, the resonances became sharpened to show 12 aromatic resonances of **(*P*)-(20,4)⊃(*P*)-(9,6)**. Spectra were measured in CD_2Cl_2 with total concentrations of **(*P*)-(20,4)** and **(*P*)-(9,6)** set at 1 mM. **b,** Reference ^{1}H NMR spectra of **(*P*)-(20,4)** and **(*P*)-(9,6)**. These spectra do not match with the spectrum shown in **a**, confirming that the i-DWNT complex of **(*P*)-(20,4)⊃(*P*)-(9,6)** were observed as an independent species. At 25 °C, the in-and-out exchange processes with **(*P*)-(20,4)⊃(*P*)-(9,6)** was slightly slower than the NMR timescale and, at –30 °C, became sufficient slow.

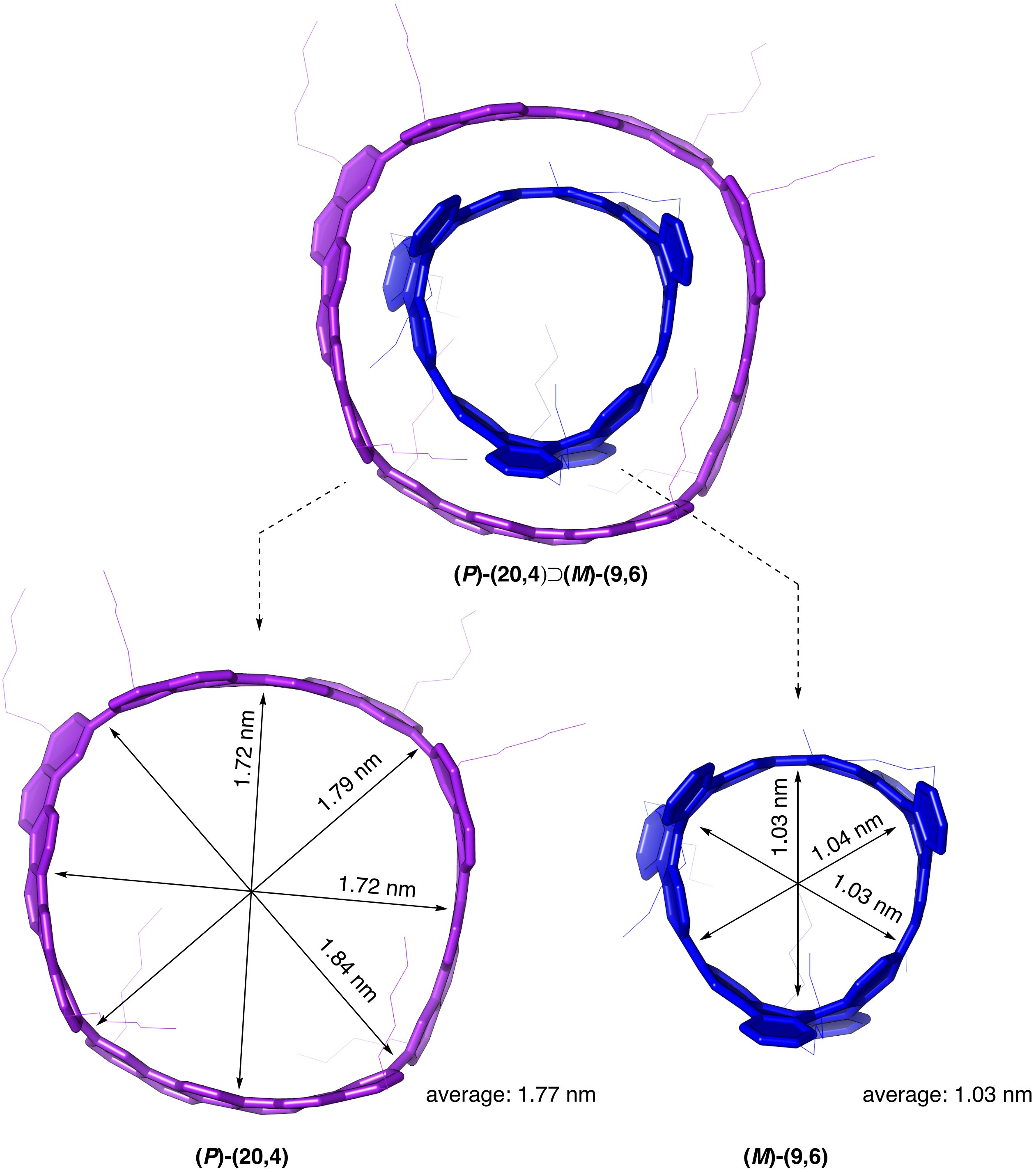

Supplementary Fig. 7 | Structural parameters from crystal structure of the i-DWNT complex of (*P*)-(20,4)⊃(*M*)-(9,6). Distances were measured between the middle points of aromatic panel or single bond linkages.

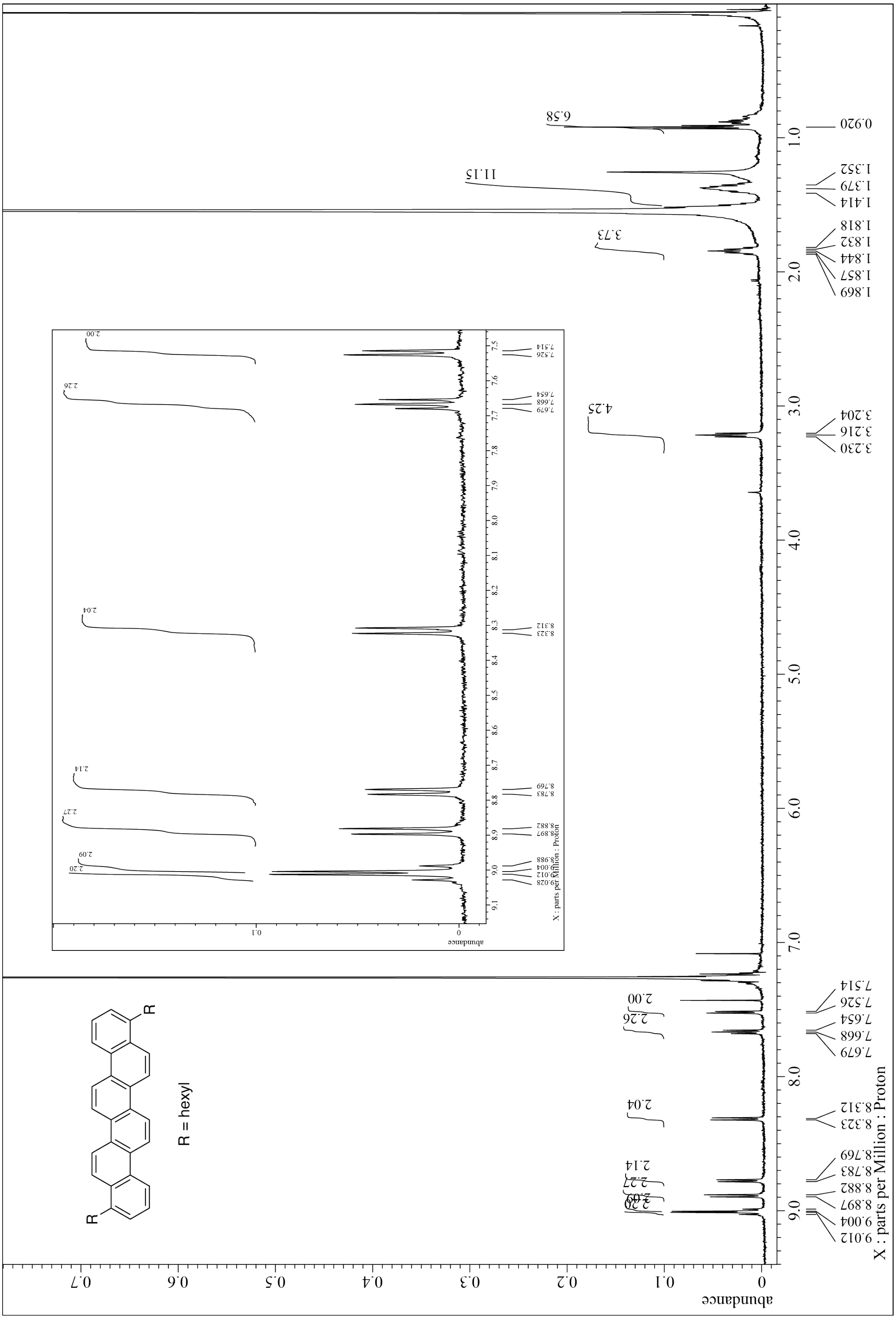

Supplementary Fig. 8 | ^{1}H NMR spectra of 1,9-dihexylfulminene (2) in $CDCl_3$ at 25 °C.

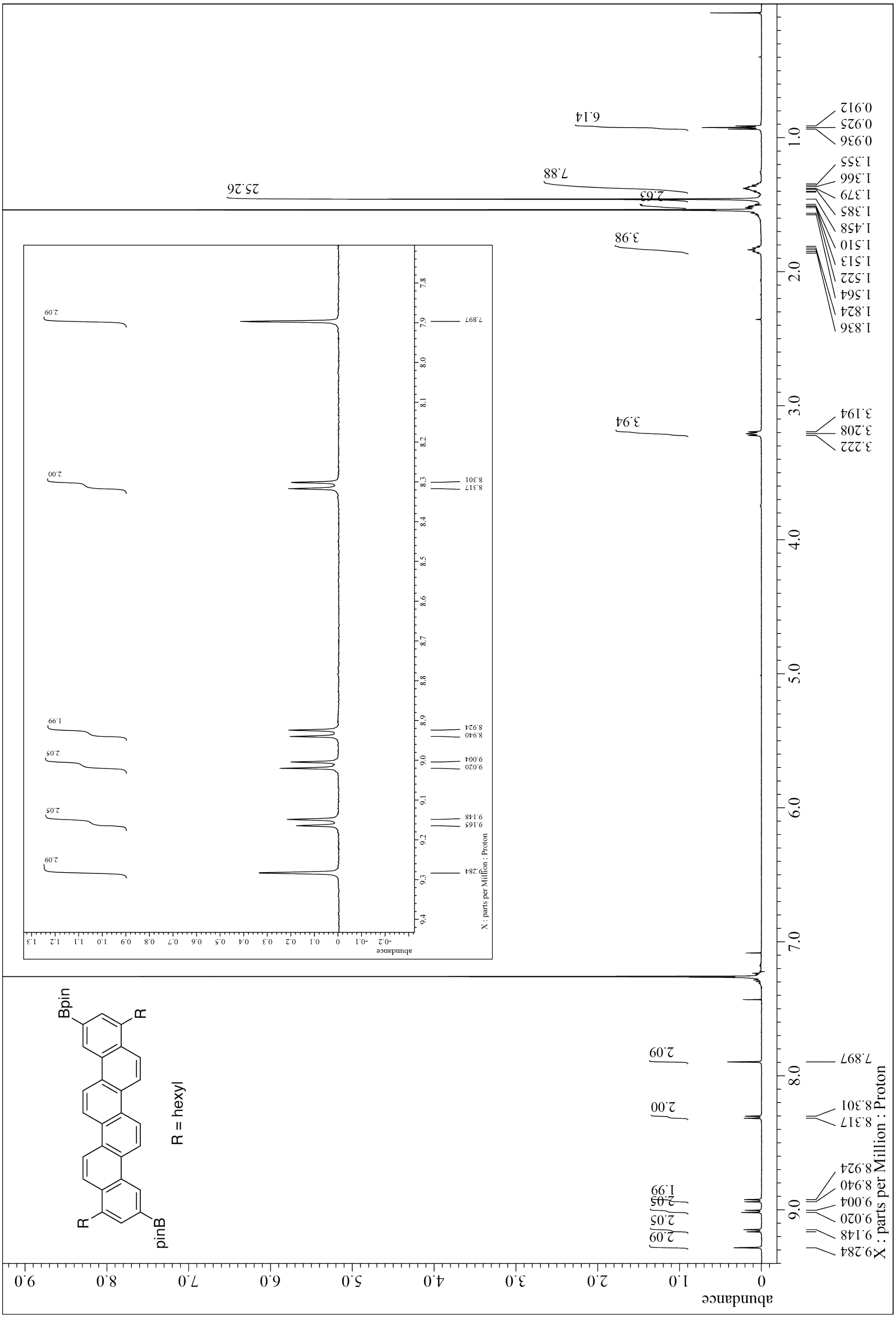

Supplementary Fig. 9 | ^{1}H NMR spectra of 3,11-bis(4,4,5,5-tetramethyl-1,3,2-dioxaborolan-2-yl)-1,9-dihexylfulminene (3) in $CDCl_3$ at 25 °C.

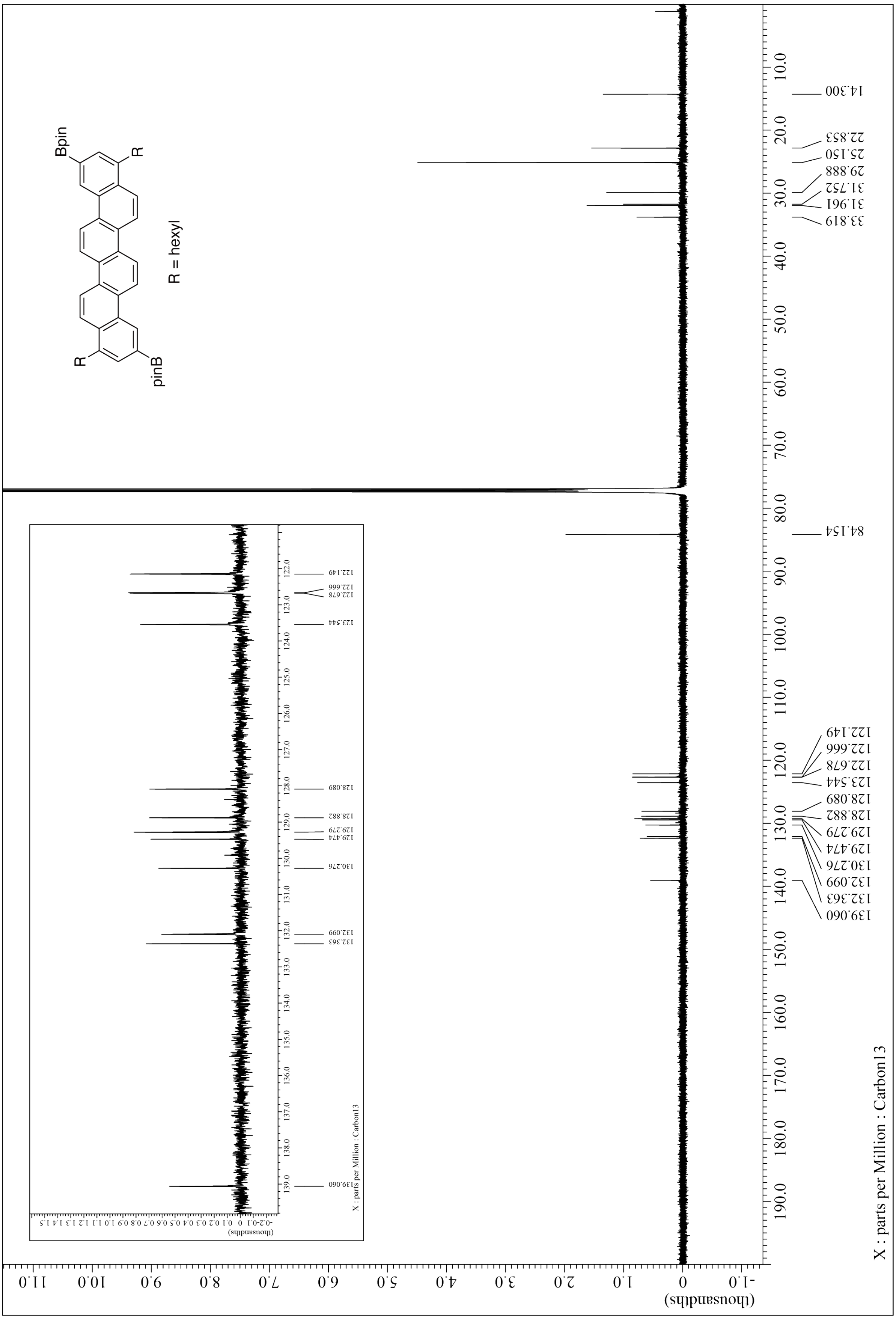

Supplementary Fig. 10 | ^{13}C NMR spectra of 3,11-bis(4,4,5,5-tetramethyl-1,3,2-dioxaborolan-2-yl)-1,9-dihexylfulminene (3) in $CDCl_3$ at 25 °C.

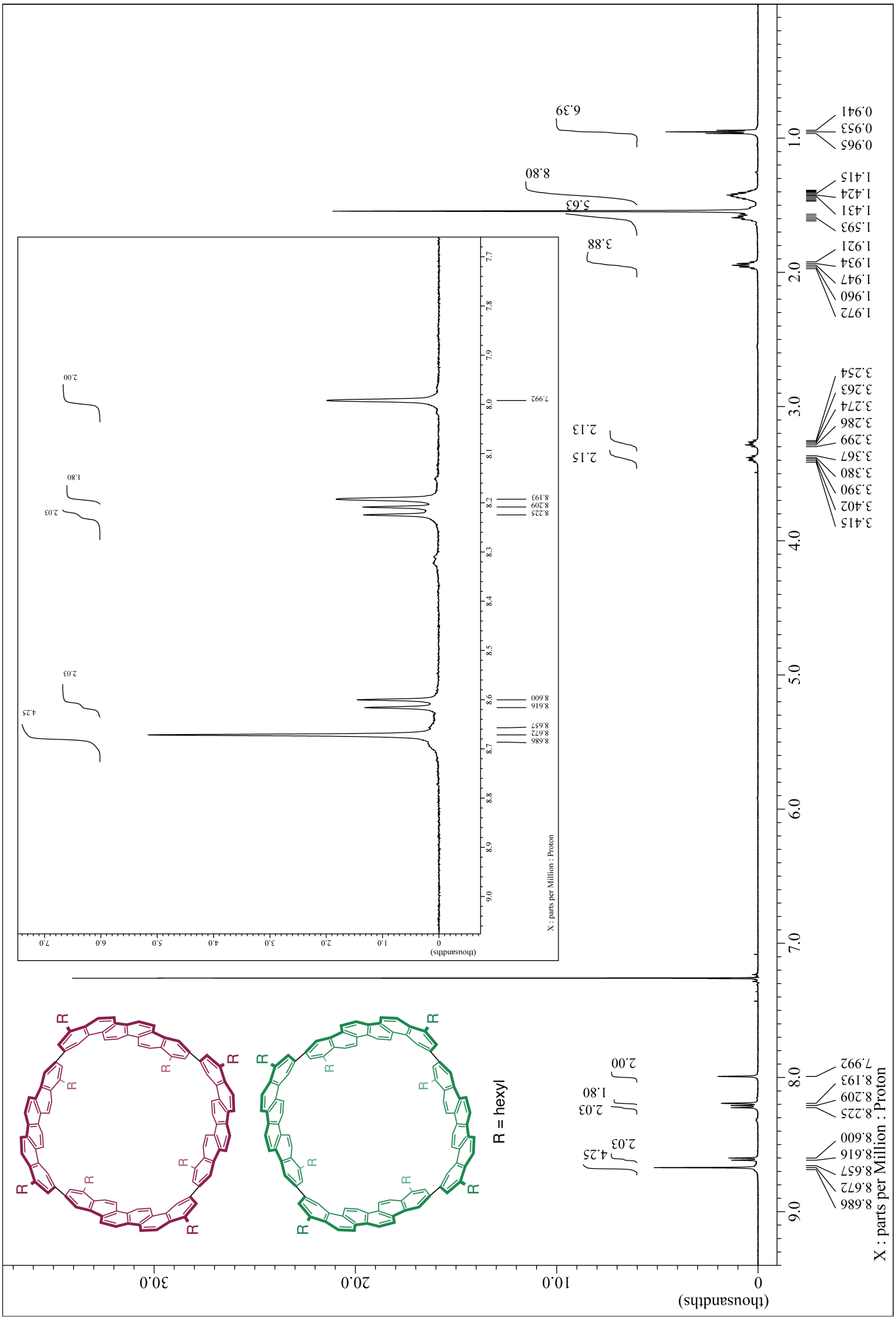

Supplementary Fig. 11 | ^{1}H NMR spectra of [4]CF in $CDCl_3$ at 25 °C.

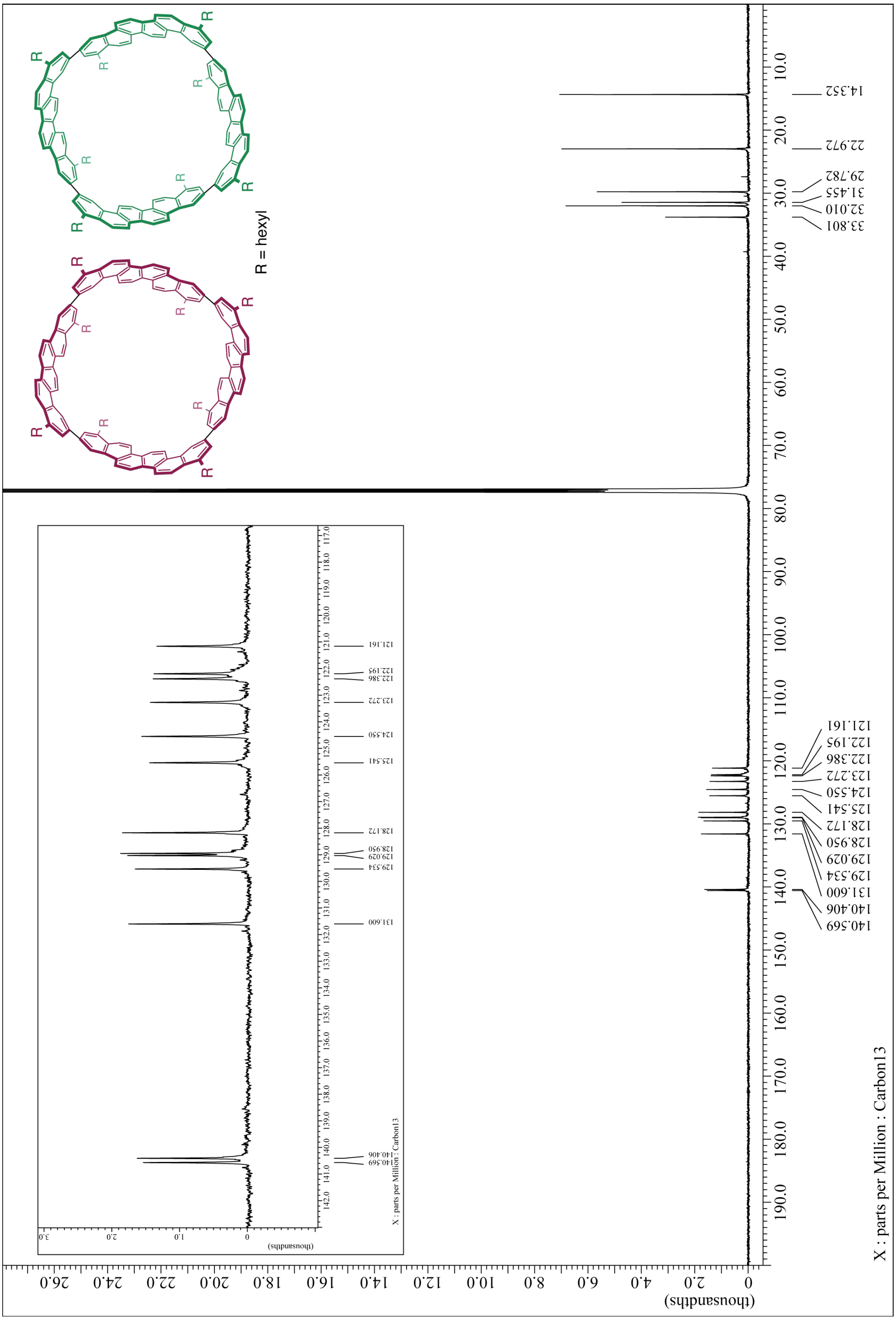

Supplementary Fig. 12 | ^{13}C NMR spectra of [4]CF in $CDCl_3$ at 25 °C.

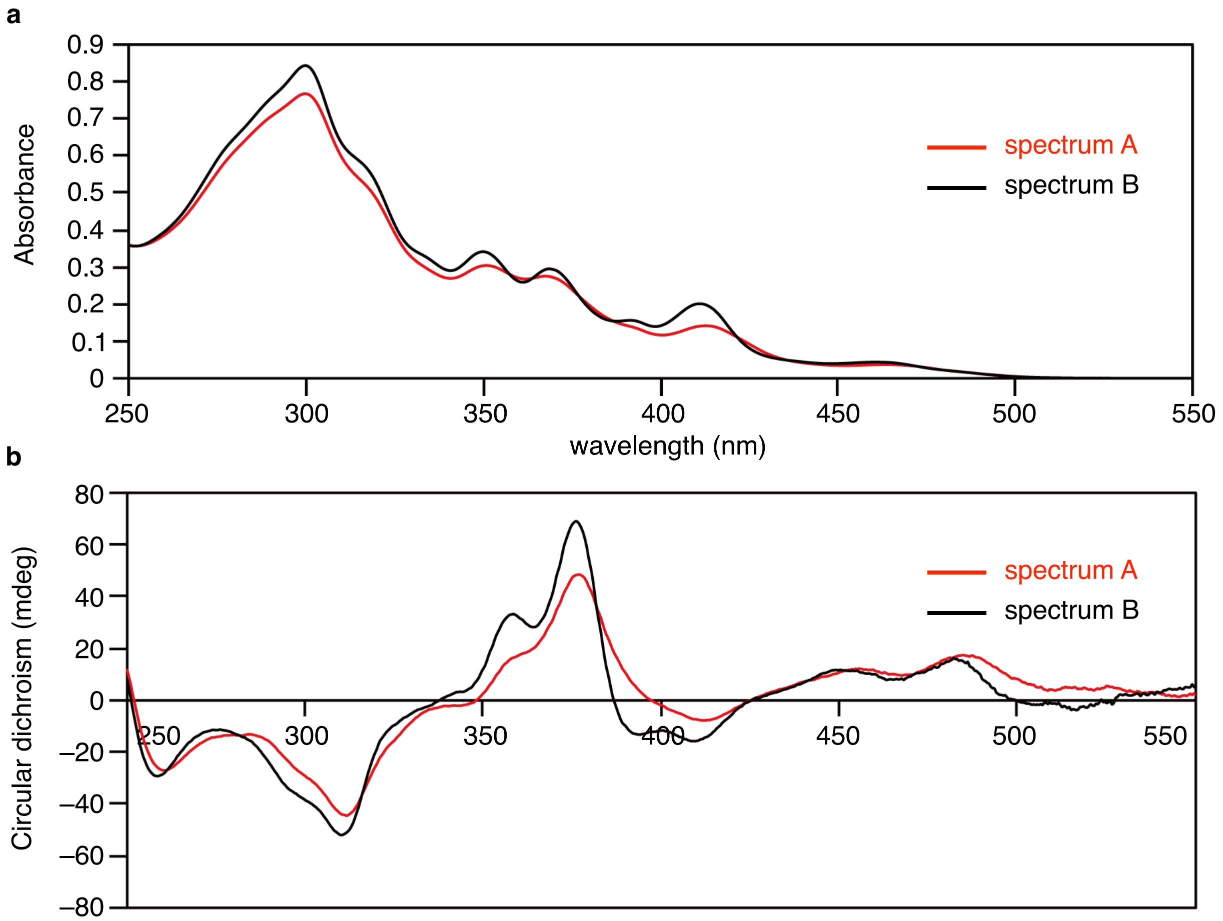

Supplementary Fig. 13 | Optical properties of i-DWNT complex. Spectra of a mixture of **(*P*)-(20,4)** and **(*M*)-(9,6)** are shown as **spectrum A**, and summed spectra of each component are shown as **spectrum B**. The spectra were measured in dichloromethane at 1.84×10^{-6} M at 25 °C. **a,** UV-vis spectra. **b,** CD spectra.

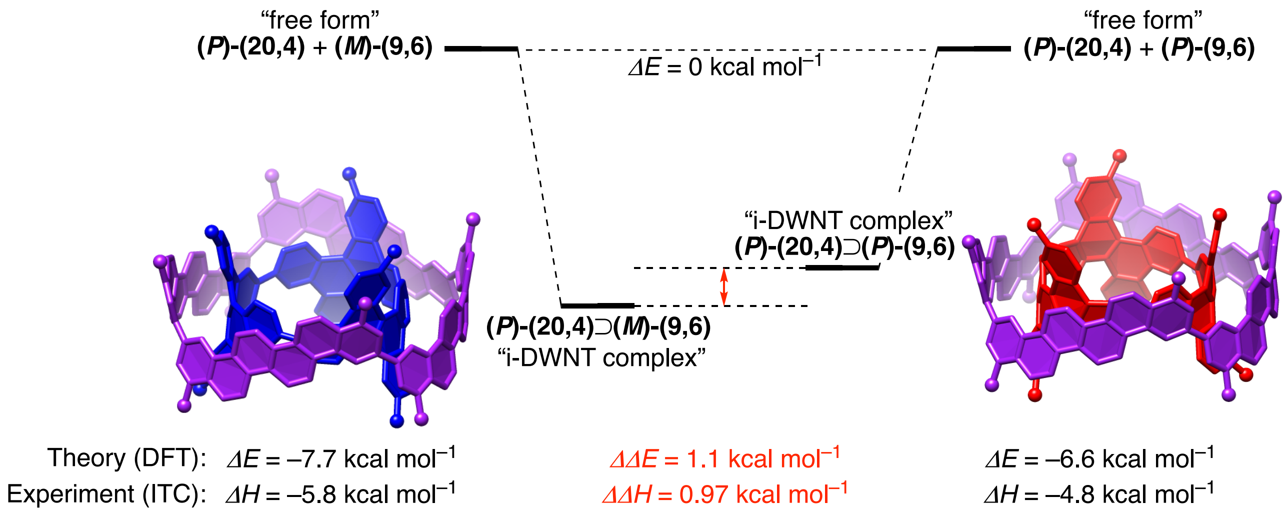

Supplementary Fig. 14 | Energetics of i-DWNT complexation from theoretical calculations. DFT calculations were performed at LC-BLYP/6-311G(d) in the presence of PCM (CH_2Cl_2) solvation with BSSE corrections. As references, experimental enthalpy values from ITC analyses are shown. Theoretical *ΔE*/*ΔΔE* values nicely correlated with experimental *ΔH*/*ΔΔH* values, which indicated a dominant role of electronic interactions to determine the stereoselectivity. Calculations were performed by adopting methyl-substituted congeners as models.

Supplementary Tables

Supplementary Table 1 | Crystal data and structure refinement for (*P*)-(20,4)⊃(*M*)-(9,6) and (*M*)-(20,4)⊃(*P*)-(9,6).

CCDC No.	2034189	
Empirical formula	$C_{268} H_{268} Cl_6$	
Formula weight	3701.51	
Temperature	100(2) K	
Wavelength	0.810 Å	
Crystal system	Monoclinic	
Space group	$P\,2_1/n$	
Unit cell dimensions	a = 26.010(5) Å	α = 90°.
	b = 31.100(6) Å	β = 90.93(3)°.
	c = 26.020(5) Å	γ = 90°.
Volume	21045(7) $Å^3$	
Z	4	
Density (calculated)	1.168 Mg/m^3	
Absorption coefficient	0.191 mm^{-1}	
F(000)	7912	
Crystal size	0.100 × 0.050 × 0.050 mm^3	
Theta range for data collection	1.163 to 29.166°.	
Index ranges	$-31<=h<=31$, $-37<=k<=37$, $-31<=l<=31$	
Reflections collected	252354	
Independent reflections	37835 [R(int) = 0.0405]	
Completeness to theta = 29.078°	98.7 %	
Absorption correction	Semi-empirical from equivalents	
Max. and min. transmission	1.000 and 0.745	
Refinement method	Full-matrix least-squares on F^2	
Data / restraints / parameters	37835 / 1624 / 3195	
Goodness-of-fit on F^2	1.341	
Final R indices [I>2sigma(I)]	R_1 = 0.0927, wR_2 = 0.2985	
R indices (all data)	R_1 = 0.1109, wR_2 = 0.3209	
Extinction coefficient	n/a	
Largest diff. peak and hole	0.606 and –0.557 e.$Å^{-3}$	

Supplementary Table 2 | Cartesian coordinates of methyl-substituted (*P*)-(20,4)-[4]CF.

SCF Done: E(RB3LYP) = -4311.71422419 A.U. after 6 cycles

Center Number	Atomic Number	Atomic Type	Coordinates (Angstroms) X	Y	Z
1	6	0	7.783612	-4.944343	-0.423233
2	6	0	6.748225	-5.718606	0.080586
3	6	0	5.968731	-6.550111	-0.755439
4	6	0	6.282873	-6.610425	-2.145663
5	6	0	7.423936	-5.908739	-2.643457
6	6	0	8.135486	-5.089167	-1.787329
7	1	0	6.455255	-5.574205	1.113572
8	6	0	4.783825	-7.228799	-0.259200
9	6	0	5.393665	-7.320742	-3.010896
10	1	0	8.978686	-4.522206	-2.172897
11	6	0	4.207317	-7.823089	-2.556822
12	6	0	3.841607	-7.755656	-1.175358
13	1	0	3.544088	-8.310760	-3.261883
14	6	0	4.508188	-7.350686	1.132747
15	6	0	3.331818	-7.879386	1.590550
16	6	0	2.285343	-8.250954	0.697009
17	6	0	2.541247	-8.175808	-0.698203
18	1	0	5.253037	-7.034896	1.854166
19	1	0	3.174798	-7.943063	2.660734
20	6	0	0.964369	-8.600969	1.174165
21	6	0	1.460110	-8.429392	-1.591754
22	6	0	0.184703	-8.621008	-1.133963
23	6	0	-0.113068	-8.667557	0.257982
24	1	0	1.626641	-8.398070	-2.661941
25	1	0	-0.612584	-8.758340	-1.855386
26	6	0	0.693229	-8.855517	2.555629
27	6	0	-0.576310	-9.074841	3.009661
28	6	0	-1.708399	-8.958511	2.144384
29	6	0	-1.476779	-8.737771	0.754184
30	1	0	1.514820	-8.906875	3.260723
31	6	0	-3.047710	-8.985807	2.642081
32	6	0	-2.582141	-8.460060	-0.081880
33	6	0	-3.871821	-8.369039	0.421877
34	6	0	-4.089470	-8.681378	1.785920
35	1	0	-2.413748	-8.180001	-1.114813
36	1	0	-5.105374	-8.660793	2.171417
37	6	0	3.871867	8.368983	0.422528
38	6	0	2.582129	8.460078	-0.081065

39	6	0	1.476866	8.737690	0.755164
40	6	0	1.708651	8.958251	2.145366
41	6	0	3.048023	8.985470	2.642908
42	6	0	4.089679	8.681143	1.786585
43	1	0	2.413611	8.180150	-1.114014
44	6	0	0.113097	8.667543	0.259113
45	6	0	0.576662	9.074471	3.010790
46	1	0	5.105628	8.660500	2.171959
47	6	0	-0.692930	8.855207	2.556879
48	6	0	-0.964231	8.600829	1.175416
49	1	0	-1.514438	8.906479	3.262075
50	6	0	-0.184842	8.621181	-1.132802
51	6	0	-1.460303	8.429623	-1.590466
52	6	0	-2.541331	8.175913	-0.696821
53	6	0	-2.285260	8.250870	0.698372
54	1	0	0.612359	8.758616	-1.854302
55	1	0	-1.626964	8.398450	-2.660638
56	6	0	-3.841745	7.755820	-1.173877
57	6	0	-3.331627	7.879177	1.591987
58	6	0	-4.508050	7.350534	1.134253
59	6	0	-4.783852	7.228836	-0.257677
60	1	0	-3.174478	7.942709	2.662161
61	1	0	-5.252812	7.034643	1.855718
62	6	0	-4.207620	7.823441	-2.555287
63	6	0	-5.394020	7.321150	-3.009289
64	6	0	-6.283122	6.610712	-2.144046
65	6	0	-5.968815	6.550211	-0.753868
66	1	0	-3.544476	8.311212	-3.260360
67	6	0	-7.424241	5.909089	-2.641801
68	6	0	-6.748207	5.718591	0.082137
69	6	0	-7.783651	4.944392	-0.421664
70	6	0	-8.135686	5.089398	-1.785699
71	1	0	-6.455114	5.574051	1.115069
72	1	0	-8.978930	4.522486	-2.171244
73	6	0	8.369072	-3.871874	0.421436
74	6	0	8.460102	-2.582131	-0.082156
75	6	0	8.737823	-1.476877	0.754048
76	6	0	8.958565	-1.708675	2.144218
77	6	0	8.985849	-3.048052	2.641744
78	6	0	8.681409	-4.089700	1.785451
79	1	0	8.180040	-2.413603	-1.115067
80	6	0	8.667611	-0.113103	0.258019
81	6	0	9.074896	-0.576695	3.009639
82	1	0	8.660817	-5.105652	2.170818

83	6	0	8.855571	0.692901	2.555768
84	6	0	8.601014	0.964216	1.174341
85	1	0	8.906932	1.514404	3.260965
86	6	0	8.621071	0.184849	-1.133888
87	6	0	8.429452	1.460314	-1.591515
88	6	0	8.175854	2.541333	-0.697826
89	6	0	8.250990	2.285249	0.697354
90	1	0	8.758414	-0.612344	-1.855413
91	1	0	8.398142	1.626986	-2.661681
92	6	0	7.755696	3.841751	-1.174816
93	6	0	7.879408	3.331605	1.591027
94	6	0	7.350702	4.508031	1.133373
95	6	0	7.228824	4.783847	-0.258540
96	1	0	7.943078	3.174447	2.661192
97	1	0	7.034900	5.252785	1.854885
98	6	0	7.823140	4.207640	-2.556231
99	6	0	7.320788	5.394042	-3.010157
100	6	0	6.610456	6.283133	-2.144815
101	6	0	6.550132	5.968811	-0.754632
102	1	0	8.310825	3.544505	-3.261373
103	6	0	5.908765	7.424254	-2.642470
104	6	0	5.718613	6.748191	0.081485
105	6	0	4.944346	7.783636	-0.422207
106	6	0	5.089179	8.135686	-1.786257
107	1	0	5.574205	6.455086	1.114432
108	1	0	4.522215	8.978932	-2.171722
109	6	0	-4.944402	-7.783588	-0.422656
110	6	0	-5.718612	-6.748210	0.081263
111	6	0	-6.550228	-5.968721	-0.754656
112	6	0	-6.610712	-6.282859	-2.144873
113	6	0	-5.909078	-7.423915	-2.642759
114	6	0	-5.089394	-8.135459	-1.786735
115	1	0	-5.574084	-6.455240	1.114231
116	6	0	-7.228864	-4.783822	-0.258329
117	6	0	-7.321144	-5.393654	-3.010015
118	6	0	-7.823443	-4.207311	-2.555875
119	6	0	-7.755839	-3.841604	-1.174419
120	1	0	-8.311206	-3.544082	-3.260873
121	6	0	-7.350582	-4.508192	1.133634
122	6	0	-7.879234	-3.331826	1.591506
123	6	0	-8.250917	-2.285350	0.698014
124	6	0	-8.175941	-2.541249	-0.697208
125	1	0	-7.034700	-5.253043	1.855011
126	1	0	-7.942782	-3.174810	2.661699

127	6	0	-8.600882	-0.964380	1.175217
128	6	0	-8.429641	-1.460111	-1.590724
129	6	0	-8.621204	-0.184706	-1.132905
130	6	0	-8.667582	0.113061	0.259047
131	1	0	-8.398455	-1.626639	-2.660915
132	1	0	-8.758628	0.612583	-1.854309
133	6	0	-8.855278	-0.693249	2.556710
134	6	0	-9.074545	0.576287	3.010776
135	6	0	-8.958309	1.708383	2.145493
136	6	0	-8.737731	1.476770	0.755266
137	1	0	-8.906562	-1.514845	3.261803
138	6	0	-8.985527	3.047693	2.643202
139	6	0	-8.460106	2.582135	-0.080823
140	6	0	-8.369011	3.871810	0.422931
141	6	0	-8.681186	4.089454	1.787012
142	1	0	-8.180166	2.413745	-1.113789
143	1	0	-8.660542	5.105355	2.172513
144	1	0	-4.522474	-8.978655	-2.172376
145	1	0	5.641101	-7.417735	-4.062257
146	1	0	0.732454	9.289803	4.062147
147	1	0	-5.641581	7.418286	-4.060607
148	1	0	-9.289890	0.731949	4.062149
149	1	0	-7.418266	-5.641087	-4.061365
150	1	0	9.290364	-0.732496	4.060966
151	1	0	7.417790	5.641616	-4.061484
152	6	0	3.329426	9.287486	4.096069
153	1	0	2.924143	10.260917	4.395045
154	1	0	2.885875	8.536799	4.760803
155	1	0	4.405428	9.303192	4.285934
156	6	0	-7.824120	6.011088	-4.095002
157	1	0	-8.009593	7.049081	-4.393968
158	1	0	-7.044975	5.619528	-4.759647
159	1	0	-8.737674	5.442413	-4.285009
160	6	0	-6.011062	-7.823619	-4.096009
161	1	0	-7.049052	-8.009057	-4.395009
162	1	0	-5.619494	-7.044395	-4.760556
163	1	0	-5.442385	-8.737151	-4.286120
164	6	0	-9.287559	3.328917	4.096394
165	1	0	-10.260997	2.923603	4.395307
166	1	0	-8.536884	2.885278	4.761083
167	1	0	-9.303262	4.404895	4.286394
168	1	0	-0.731975	-9.290329	4.061006
169	6	0	-3.328938	-9.288016	4.095236
170	1	0	-2.923586	-10.261468	4.394047

171	1	0	-2.885340	-8.537394	4.760012
172	1	0	-4.404918	-9.303783	4.285221
173	6	0	7.823644	-6.010545	-4.096719
174	1	0	8.009074	-7.048499	-4.395848
175	1	0	7.044425	-5.618888	-4.761220
176	1	0	8.737181	-5.441852	-4.286756
177	6	0	9.288055	-3.329469	4.094863
178	1	0	10.261524	-2.924187	4.393716
179	1	0	8.537454	-2.885927	4.759700
180	1	0	9.303789	-4.405473	4.284715
181	6	0	6.010579	7.824148	-4.095679
182	1	0	7.048534	8.009628	-4.394775
183	1	0	5.618938	7.045010	-4.760283
184	1	0	5.441878	8.737703	-4.285605

Supplementary Table 3 | Cartesian coordinates of methyl-substituted (*P*)-(17,3)-[4]CF.

SCF Done: E(RB3LYP) = -4311.69779285   A.U. after   6 cycles

Center Number	Atomic Number	Atomic Type	Coordinates (Angstroms) X	Y	Z
1	6	0	6.643811	-5.589826	1.874622
2	6	0	7.181110	-4.309513	1.904787
3	6	0	7.978049	-3.817385	0.847002
4	6	0	8.245777	-4.665275	-0.266800
5	6	0	7.851694	-6.038230	-0.209907
6	6	0	7.063459	-6.466562	0.841678
7	1	0	6.841913	-3.619377	2.668083
8	6	0	8.366252	-2.420102	0.790219
9	6	0	8.811070	-4.079514	-1.442818
10	1	0	6.717204	-7.496560	0.854657
11	6	0	8.955676	-2.724671	-1.559677
12	6	0	8.698076	-1.847159	-0.459664
13	1	0	9.318597	-2.311978	-2.494705
14	6	0	8.381532	-1.585537	1.943908
15	6	0	8.624464	-0.240593	1.851312
16	6	0	8.729111	0.402663	0.583369
17	6	0	8.728873	-0.406536	-0.583404
18	1	0	8.226306	-2.029150	2.921471
19	1	0	8.635698	0.354278	2.757534
20	6	0	8.698975	1.843296	0.459623
21	6	0	8.624421	0.236764	-1.851339
22	6	0	8.382124	1.581824	-1.943926

23	6	0	8.367338	2.416400	-0.790240
24	1	0	8.635268	-0.358124	-2.757555
25	1	0	8.227025	2.025497	-2.921481
26	6	0	8.957057	2.720694	1.559612
27	6	0	8.813103	4.075610	1.442755
28	6	0	8.248025	4.661642	0.266772
29	6	0	7.979814	3.813869	-0.847003
30	1	0	9.319823	2.307843	2.494630
31	6	0	7.854605	6.034792	0.209886
32	6	0	7.183063	4.306381	-1.904755
33	6	0	6.646401	5.586959	-1.874583
34	6	0	7.066521	6.463495	-0.841659
35	1	0	6.843491	3.616404	-2.668027
36	1	0	6.720747	7.493654	-0.854618
37	6	0	-6.463811	-5.613989	-2.561321
38	6	0	-6.818287	-4.625197	-1.652806
39	6	0	-7.360657	-3.388426	-2.071581
40	6	0	-7.568158	-3.166462	-3.466141
41	6	0	-7.318364	-4.223443	-4.391972
42	6	0	-6.773502	-5.405003	-3.929791
43	1	0	-6.607114	-4.785848	-0.602425
44	6	0	-7.614694	-2.305533	-1.135516
45	6	0	-7.961670	-1.863232	-3.899364
46	1	0	-6.507524	-6.170897	-4.651575
47	6	0	-8.041906	-0.816365	-3.026643
48	6	0	-7.839666	-0.994288	-1.622401
49	1	0	-8.305371	0.162872	-3.408989
50	6	0	-7.621428	-2.507182	0.274135
51	6	0	-7.778994	-1.467356	1.150232
52	6	0	-7.848866	-0.116931	0.699750
53	6	0	-7.848796	0.120308	-0.699858
54	1	0	-7.528015	-3.511605	0.670994
55	1	0	-7.780805	-1.681629	2.212333
56	6	0	-7.839254	0.997659	1.622295
57	6	0	-7.778300	1.470702	-1.150336
58	6	0	-7.620287	2.510456	-0.274234
59	6	0	-7.613679	2.308804	1.135418
60	1	0	-7.779987	1.684977	-2.212437
61	1	0	-7.526410	3.514837	-0.671090
62	6	0	-8.041604	0.819821	3.026531
63	6	0	-7.960913	1.866648	3.899257
64	6	0	-7.566805	3.169702	3.466047
65	6	0	-7.359176	3.391580	2.071493
66	1	0	-8.305516	-0.159299	3.408867

67	6	0	-7.316557	4.226565	4.391890
68	6	0	-6.816241	4.628111	1.652737
69	6	0	-6.461338	5.616738	2.561265
70	6	0	-6.771153	5.407881	3.929727
71	1	0	-6.604980	4.788674	0.602360
72	1	0	-6.504849	6.173650	4.651522
73	6	0	5.488017	-5.911207	2.750323
74	6	0	4.397370	-6.581771	2.212219
75	6	0	3.137100	-6.580693	2.851111
76	6	0	2.990947	-5.877902	4.082584
77	6	0	4.149378	-5.333136	4.719268
78	6	0	5.357720	-5.356229	4.048897
79	1	0	4.474641	-6.976183	1.206192
80	6	0	1.963837	-7.140936	2.205187
81	6	0	1.670968	-5.670915	4.591508
82	1	0	6.226751	-4.898388	4.513741
83	6	0	0.566275	-6.023003	3.866774
84	6	0	0.676078	-6.741859	2.635060
85	1	0	-0.416622	-5.793283	4.262867
86	6	0	2.060388	-8.066345	1.127253
87	6	0	0.947823	-8.525455	0.473994
88	6	0	-0.346776	-8.001140	0.758149
89	6	0	-0.473489	-7.064294	1.817711
90	1	0	3.034227	-8.446290	0.838719
91	1	0	1.072903	-9.241376	-0.330128
92	6	0	-1.497754	-8.328527	-0.054783
93	6	0	-1.721972	-6.396220	1.979277
94	6	0	-2.745256	-6.572372	1.085894
95	6	0	-2.658854	-7.523301	0.029278
96	1	0	-1.843172	-5.671122	2.775850
97	1	0	-3.657105	-5.999520	1.212804
98	6	0	-1.509533	-9.444959	-0.948625
99	6	0	-2.590827	-9.718115	-1.738585
100	6	0	-3.705475	-8.826041	-1.809185
101	6	0	-3.714845	-7.684191	-0.955025
102	1	0	-0.664680	-10.124294	-0.963159
103	6	0	-4.767406	-9.006587	-2.747530
104	6	0	-4.685716	-6.680312	-1.170920
105	6	0	-5.644557	-6.790494	-2.170354
106	6	0	-5.703812	-8.002891	-2.904595
107	1	0	-4.599858	-5.744938	-0.631221
108	1	0	-6.495855	-8.138142	-3.635447
109	6	0	5.490743	5.908908	-2.750259
110	6	0	4.400384	6.579911	-2.212117

111	6	0	3.140118	6.579427	-2.851020
112	6	0	2.993671	5.876781	-4.082541
113	6	0	4.151868	5.331558	-4.719255
114	6	0	5.360215	5.354080	-4.048872
115	1	0	4.477820	6.974223	-1.206063
116	6	0	1.967091	7.140144	-2.205074
117	6	0	1.673606	5.670400	-4.591489
118	6	0	0.569060	6.022915	-3.866740
119	6	0	0.679162	6.741649	-2.634981
120	1	0	-0.413933	5.793652	-4.262860
121	6	0	2.064029	8.065469	-1.127102
122	6	0	0.951655	8.525034	-0.473837
123	6	0	-0.343168	8.001288	-0.758025
124	6	0	-0.470274	7.064543	-1.817629
125	1	0	3.038027	8.444990	-0.838549
126	1	0	1.077036	9.240876	0.330309
127	6	0	-1.494016	8.329146	0.054900
128	6	0	-1.719048	6.397028	-1.979247
129	6	0	-2.742271	6.573592	-1.085875
130	6	0	-2.655469	7.524435	-0.029214
131	1	0	-1.840556	5.672024	-2.775859
132	1	0	-3.654374	6.001155	-1.212834
133	6	0	-1.505326	9.445556	0.948777
134	6	0	-2.586523	9.719170	1.738710
135	6	0	-3.701570	8.827590	1.809253
136	6	0	-3.711415	7.685760	0.955069
137	1	0	-0.660175	10.124518	0.963355
138	6	0	-4.763451	9.008589	2.747564
139	6	0	-4.682732	6.682302	1.170922
140	6	0	-5.641558	6.792889	2.170326
141	6	0	-5.700306	8.005303	2.904579
142	1	0	-4.597263	5.746899	0.631212
143	1	0	-6.492314	8.140894	3.635406
144	1	0	6.229051	4.895894	-4.513740
145	1	0	9.062104	-4.714684	-2.285316
146	1	0	-8.156835	-1.691156	-4.951997
147	1	0	-8.156175	1.694655	4.951886
148	1	0	-2.575359	10.605500	2.363543
149	1	0	1.544086	5.168173	-5.543941
150	1	0	1.541657	-5.168565	5.543924
151	1	0	-2.580036	-10.604465	-2.363396
152	6	0	-7.575736	-4.039792	-5.869212
153	1	0	-8.620388	-3.775599	-6.069661
154	1	0	-6.954894	-3.242807	-6.295088

155	1	0	-7.354665	-4.959132	-6.416919
156	6	0	-7.574043	4.043021	5.869122
157	1	0	-8.618818	3.779295	6.069548
158	1	0	-6.953568	3.245755	6.295008
159	1	0	-7.352571	4.962258	6.416840
160	6	0	4.052587	4.676883	-6.076980
161	1	0	3.420768	3.781074	-6.050310
162	1	0	3.620803	5.352709	-6.823761
163	1	0	5.040156	4.373686	-6.433252
164	6	0	-4.842426	10.255250	3.597056
165	1	0	-3.984322	10.339908	4.274487
166	1	0	-4.859680	11.164266	2.985285
167	1	0	-5.747165	10.248926	4.209895
168	1	0	9.064512	4.710650	2.285238
169	6	0	8.224839	6.992752	1.317641
170	1	0	7.758530	6.712274	2.269670
171	1	0	9.307291	7.020719	1.485955
172	1	0	7.898555	8.007468	1.076647
173	6	0	8.221313	-6.996318	-1.317757
174	1	0	7.754861	-6.715615	-2.269659
175	1	0	9.303703	-7.024703	-1.486351
176	1	0	7.894675	-8.010908	-1.076715
177	6	0	4.050371	-4.678321	6.076947
178	1	0	3.418956	-3.782229	6.050201
179	1	0	3.618270	-5.353898	6.823769
180	1	0	5.038070	-4.375543	6.433213
181	6	0	-4.846900	-10.253228	-3.597004
182	1	0	-3.988811	-10.338276	-4.274404
183	1	0	-4.864576	-11.162226	-2.985219
184	1	0	-5.751615	-10.246516	-4.209873

Supplementary Table 4 | Cartesian coordinates of methyl-substituted (10,10)$_{AABB}$-[4]CF.

SCF Done: E(RB3LYP) = -4311.68232365 A.U. after 6 cycles

Center Number	Atomic Number	Atomic Type	Coordinates (Angstroms) X	Y	Z
1	6	0	-10.641694	-0.630111	-0.391440
2	6	0	-9.767551	-1.492260	0.262967
3	6	0	-9.468855	-2.777851	-0.244818
4	6	0	-10.121730	-3.216044	-1.436397
5	6	0	-11.135829	-2.401184	-2.019840
6	6	0	-11.361005	-1.141535	-1.503316

7	1	0	-9.227508	-1.134538	1.131324
8	6	0	-8.422669	-3.608365	0.332567
9	6	0	-9.679815	-4.422918	-2.059395
10	1	0	-12.059658	-0.496371	-2.024290
11	6	0	-8.574479	-5.086604	-1.611869
12	6	0	-7.888373	-4.678143	-0.426190
13	1	0	-8.240973	-5.970569	-2.143091
14	6	0	-7.879732	-3.373287	1.628477
15	6	0	-6.835150	-4.113799	2.118267
16	6	0	-6.160128	-5.073986	1.309054
17	6	0	-6.668509	-5.320793	0.007017
18	1	0	-8.322579	-2.612371	2.261804
19	1	0	-6.470471	-3.902700	3.117129
20	6	0	-4.943631	-5.729898	1.740309
21	6	0	-5.902524	-6.135775	-0.876041
22	6	0	-4.664978	-6.599562	-0.522130
23	6	0	-4.137821	-6.393726	0.783923
24	1	0	-6.265585	-6.331363	-1.878054
25	1	0	-4.092796	-7.172904	-1.242734
26	6	0	-4.518421	-5.741897	3.105720
27	6	0	-3.353617	-6.348426	3.488302
28	6	0	-2.438530	-6.879156	2.527036
29	6	0	-2.808804	-6.844711	1.151865
30	1	0	-5.158353	-5.309131	3.866621
31	6	0	-1.150204	-7.382909	2.887742
32	6	0	-1.833758	-7.142429	0.173385
33	6	0	-0.535658	-7.498988	0.514664
34	6	0	-0.242984	-7.687143	1.891390
35	1	0	-2.070329	-6.958960	-0.867095
36	1	0	0.738981	-8.052910	2.176741
37	6	0	0.461256	7.384091	-0.584757
38	6	0	1.819142	7.134660	-0.417797
39	6	0	2.635998	6.703889	-1.486758
40	6	0	2.046816	6.489580	-2.764809
41	6	0	0.711836	6.947641	-2.990301
42	6	0	-0.035724	7.390999	-1.917482
43	1	0	2.241510	7.110503	0.578303
44	6	0	4.020350	6.326360	-1.273834
45	6	0	2.786263	5.749271	-3.738209
46	1	0	-1.059792	7.698054	-2.103444
47	6	0	3.987517	5.172010	-3.426671
48	6	0	4.638057	5.433475	-2.180567
49	1	0	4.489136	4.560221	-4.168734
50	6	0	4.785145	6.824627	-0.181212

51	6	0	6.085500	6.438281	0.014256
52	6	0	6.669098	5.398300	-0.766148
53	6	0	5.904648	4.835429	-1.821253
54	1	0	4.347017	7.568112	0.476251
55	1	0	6.646431	6.869791	0.835814
56	6	0	7.967624	4.845557	-0.453691
57	6	0	6.372749	3.634953	-2.430110
58	6	0	7.482381	2.985156	-1.954445
59	6	0	8.306508	3.564154	-0.947980
60	1	0	5.793141	3.177338	-3.224211
61	1	0	7.770420	2.035843	-2.393380
62	6	0	8.936638	5.555208	0.322182
63	6	0	10.161209	5.013240	0.600058
64	6	0	10.458450	3.652055	0.280634
65	6	0	9.481415	2.891287	-0.424653
66	1	0	8.724736	6.572023	0.634524
67	6	0	11.658584	3.007104	0.708232
68	6	0	9.613832	1.485488	-0.475455
69	6	0	10.694432	0.829990	0.104628
70	6	0	11.756414	1.633658	0.600266
71	1	0	8.775739	0.906508	-0.844224
72	1	0	12.664631	1.154869	0.952396
73	6	0	-10.694427	0.829856	-0.104534
74	6	0	-9.613861	1.485399	0.475560
75	6	0	-9.481481	2.891202	0.424719
76	6	0	-10.458516	3.651913	-0.280630
77	6	0	-11.658618	3.006916	-0.708245
78	6	0	-11.756413	1.633472	-0.600242
79	1	0	-8.775762	0.906447	0.844361
80	6	0	-8.306593	3.564122	0.948034
81	6	0	-10.161298	5.013090	-0.600112
82	1	0	-12.664591	1.154635	-0.952407
83	6	0	-8.936745	5.555101	-0.322237
84	6	0	-7.967730	4.845507	0.453686
85	1	0	-8.724856	6.571903	-0.634630
86	6	0	-7.482464	2.985192	1.954539
87	6	0	-6.372851	3.635037	2.430182
88	6	0	-5.904767	4.835489	1.821268
89	6	0	-6.669218	5.398291	0.766127
90	1	0	-7.770487	2.035895	2.393518
91	1	0	-5.793241	3.177475	3.224313
92	6	0	-4.638186	5.433576	2.180556
93	6	0	-6.085635	6.438240	-0.014329
94	6	0	-4.785286	6.824616	0.181122

95	6	0	-4.020487	6.326417	1.273773
96	1	0	-6.646573	6.869699	-0.835910
97	1	0	-4.347167	7.568071	-0.476381
98	6	0	-3.987648	5.172196	3.426679
99	6	0	-2.786403	5.749491	3.738188
100	6	0	-2.046958	6.489746	2.764745
101	6	0	-2.636138	6.703972	1.486678
102	1	0	-4.489264	4.560447	4.168777
103	6	0	-0.711983	6.947833	2.990213
104	6	0	-1.819281	7.134679	0.417693
105	6	0	-0.461397	7.384125	0.584640
106	6	0	0.035578	7.391125	1.917367
107	1	0	-2.241646	7.110459	-0.578407
108	1	0	1.059643	7.698204	2.103313
109	6	0	0.535801	-7.498966	-0.514737
110	6	0	1.833891	-7.142389	-0.173444
111	6	0	2.808938	-6.844640	-1.151911
112	6	0	2.438680	-6.879080	-2.527087
113	6	0	1.150366	-7.382859	-2.887808
114	6	0	0.243145	-7.687123	-1.891466
115	1	0	2.070455	-6.958935	0.867041
116	6	0	4.137945	-6.393643	-0.783949
117	6	0	3.353772	-6.348324	-3.488335
118	6	0	4.518566	-5.741786	-3.105734
119	6	0	4.943764	-5.729802	-1.740319
120	1	0	5.158497	-5.308997	-3.866622
121	6	0	4.665078	-6.599474	0.522114
122	6	0	5.902614	-6.135678	0.876048
123	6	0	6.668610	-5.320694	-0.006998
124	6	0	6.160250	-5.073886	-1.309043
125	1	0	4.092884	-7.172815	1.242710
126	1	0	6.265657	-6.331256	1.878070
127	6	0	7.888460	-4.678038	0.426237
128	6	0	6.835280	-4.113689	-2.118237
129	6	0	7.879849	-3.373172	-1.628424
130	6	0	8.422763	-3.608253	-0.332506
131	1	0	6.470617	-3.902586	-3.117105
132	1	0	8.322700	-2.612248	-2.261739
133	6	0	8.574534	-5.086493	1.611936
134	6	0	9.679851	-4.422796	2.059494
135	6	0	10.121777	-3.215924	1.436503
136	6	0	9.468934	-2.777735	0.244906
137	1	0	8.241010	-5.970453	2.143155
138	6	0	11.135852	-2.401050	2.019968

139	6	0	9.767636	-1.492146	-0.262876
140	6	0	10.641755	-0.629981	0.391544
141	6	0	11.361029	-1.141398	1.503449
142	1	0	9.227598	-1.134432	-1.131241
143	1	0	12.059657	-0.496236	2.024456
144	1	0	-0.738804	-8.052928	-2.176826
145	1	0	-10.198482	-4.787025	-2.939166
146	1	0	2.357064	5.586717	-4.720970
147	1	0	10.899108	5.608357	1.126995
148	1	0	10.198490	-4.786892	2.939288
149	1	0	3.095692	-6.382026	-4.541214
150	1	0	-10.899195	5.608163	-1.127102
151	1	0	-2.357207	5.587002	4.720961
152	6	0	0.095937	6.883573	-4.368053
153	1	0	0.712155	7.398753	-5.113309
154	1	0	-0.024422	5.848472	-4.709699
155	1	0	-0.893225	7.348100	-4.372562
156	6	0	12.785139	3.800048	1.327885
157	1	0	13.110696	4.620254	0.678450
158	1	0	12.488348	4.245950	2.284873
159	1	0	13.650493	3.160116	1.517499
160	6	0	0.761022	-7.540517	-4.338792
161	1	0	0.728167	-6.574516	-4.856673
162	1	0	1.470168	-8.173543	-4.883991
163	1	0	-0.228550	-7.995633	-4.426996
164	6	0	11.897635	-2.861139	3.240790
165	1	0	11.237082	-3.003255	4.104252
166	1	0	12.407472	-3.815252	3.065238
167	1	0	12.655778	-2.126319	3.522508
168	1	0	-3.095528	-6.382147	4.541179
169	6	0	-0.760847	-7.540544	4.338724
170	1	0	-0.728080	-6.574545	4.856614
171	1	0	-1.469941	-8.173638	4.883914
172	1	0	0.228763	-7.995576	4.426925
173	6	0	-11.897642	-2.861283	-3.240639
174	1	0	-11.237107	-3.003446	-4.104106
175	1	0	-12.407507	-3.815374	-3.065049
176	1	0	-12.655767	-2.126447	-3.522365
177	6	0	-12.785165	3.799808	-1.327982
178	1	0	-13.110774	4.620031	-0.678596
179	1	0	-12.488340	4.245680	-2.284975
180	1	0	-13.650492	3.159846	-1.517612
181	6	0	-0.096090	6.883861	4.367971
182	1	0	-0.712318	7.399079	5.113192

183	1	0	0.024283	5.848783	4.709683
184	1	0	0.893066	7.348402	4.372455

Supplementary Table 5 | Cartesian coordinates of methyl-substituted (10,10)$_{ABAB}$-[4]CF.

SCF Done: E(RB3LYP) = -4311.68564499 A.U. after 7 cycles

Center Number	Atomic Number	Atomic Type	Coordinates (Angstroms) X	Y	Z
1	6	0	6.724343	1.558092	-4.181654
2	6	0	6.507388	2.516041	-3.197711
3	6	0	5.633902	3.607622	-3.406913
4	6	0	4.992072	3.742981	-4.674638
5	6	0	5.351138	2.863972	-5.739537
6	6	0	6.189278	1.799146	-5.473354
7	1	0	6.934658	2.363411	-2.213297
8	6	0	5.258809	4.503599	-2.324882
9	6	0	3.938226	4.698809	-4.809839
10	1	0	6.368894	1.073963	-6.260730
11	6	0	3.454895	5.376478	-3.727334
12	6	0	4.075376	5.270891	-2.443579
13	1	0	2.613364	6.047454	-3.857913
14	6	0	6.014848	4.618492	-1.122989
15	6	0	5.589541	5.396670	-0.077366
16	6	0	4.305618	6.016427	-0.089713
17	6	0	3.523832	5.908228	-1.269613
18	1	0	6.968437	4.107578	-1.043854
19	1	0	6.209376	5.465267	0.809605
20	6	0	3.746302	6.651945	1.084021
21	6	0	2.166735	6.341037	-1.226492
22	6	0	1.590011	6.749108	-0.054082
23	6	0	2.352519	6.889860	1.139486
24	1	0	1.549711	6.263192	-2.114048
25	1	0	0.536976	7.007809	-0.045491
26	6	0	4.544048	7.049747	2.202898
27	6	0	3.984071	7.606721	3.319975
28	6	0	2.565178	7.676922	3.484715
29	6	0	1.739981	7.253660	2.403292
30	1	0	5.623348	6.960299	2.144494
31	6	0	1.950828	8.074159	4.712511
32	6	0	0.360219	7.056840	2.631780
33	6	0	-0.217521	7.289162	3.873559
34	6	0	0.592286	7.879662	4.879007

35	1	0	-0.227160	6.567396	1.864742
36	1	0	0.140457	8.152309	5.828529
37	6	0	0.217513	-7.289213	3.873495
38	6	0	-0.360238	-7.056893	2.631720
39	6	0	-1.740007	-7.253676	2.403256
40	6	0	-2.565198	-7.676903	3.484699
41	6	0	-1.950844	-8.074144	4.712486
42	6	0	-0.592293	-7.879674	4.878963
43	1	0	0.227148	-6.567479	1.864667
44	6	0	-2.352557	-6.889865	1.139459
45	6	0	-3.984093	-7.606656	3.319982
46	1	0	-0.140458	-8.152316	5.828484
47	6	0	-4.544073	-7.049678	2.202909
48	6	0	-3.746333	-6.651913	1.084013
49	1	0	-5.623371	-6.960192	2.144529
50	6	0	-1.590059	-6.749124	-0.054117
51	6	0	-2.166789	-6.341044	-1.226520
52	6	0	-3.523879	-5.908214	-1.269627
53	6	0	-4.305653	-6.016391	-0.089716
54	1	0	-0.537026	-7.007838	-0.045537
55	1	0	-1.549772	-6.263200	-2.114082
56	6	0	-4.075428	-5.270878	-2.443592
57	6	0	-5.589563	-5.396608	-0.077357
58	6	0	-6.014866	-4.618426	-1.122978
59	6	0	-5.258842	-4.503558	-2.324882
60	1	0	-6.209392	-5.465190	0.809619
61	1	0	-6.968445	-4.107492	-1.043833
62	6	0	-3.454973	-5.376494	-3.727357
63	6	0	-3.938310	-4.698829	-4.809863
64	6	0	-4.992132	-3.742976	-4.674655
65	6	0	-5.633933	-3.607585	-3.406918
66	1	0	-2.613462	-6.047493	-3.857941
67	6	0	-5.351205	-2.863977	-5.739560
68	6	0	-6.507391	-2.515983	-3.197712
69	6	0	-6.724345	-1.558045	-4.181667
70	6	0	-6.189317	-1.799130	-5.473375
71	1	0	-6.934645	-2.363330	-2.213295
72	1	0	-6.368943	-1.073956	-6.260758
73	6	0	7.289299	0.217543	-3.873517
74	6	0	7.056967	-0.360206	-2.631741
75	6	0	7.253774	-1.739968	-2.403266
76	6	0	7.677031	-2.565161	-3.484698
77	6	0	8.074286	-1.950804	-4.712480
78	6	0	7.879801	-0.592257	-4.878965

79	1	0	6.567524	0.227171	-1.864701
80	6	0	6.889961	-2.352516	-1.139468
81	6	0	7.606797	-3.984055	-3.319975
82	1	0	8.152462	-0.140422	-5.828480
83	6	0	7.049802	-4.544034	-2.202910
84	6	0	6.652011	-3.746292	-1.084024
85	1	0	6.960327	-5.623333	-2.144523
86	6	0	6.749229	-1.590020	0.054110
87	6	0	6.341141	-2.166751	1.226511
88	6	0	5.908294	-3.523836	1.269611
89	6	0	6.016476	-4.305609	0.089699
90	1	0	7.007960	-0.536993	0.045532
91	1	0	6.263312	-1.549739	2.114077
92	6	0	5.270944	-4.075379	2.443568
93	6	0	5.396686	-5.589516	0.077336
94	6	0	4.618494	-6.014814	1.122951
95	6	0	4.503616	-5.258786	2.324854
96	1	0	5.465267	-6.209342	-0.809643
97	1	0	4.107556	-6.968390	1.043801
98	6	0	5.376556	-3.454927	3.727335
99	6	0	4.698873	-3.938254	4.809832
100	6	0	3.743006	-4.992065	4.674614
101	6	0	3.607629	-5.633871	3.406880
102	1	0	6.047568	-2.613428	3.857932
103	6	0	2.863990	-5.351115	5.739512
104	6	0	2.516021	-6.507328	3.197670
105	6	0	1.558076	-6.724275	4.181617
106	6	0	1.799145	-6.189230	5.473323
107	1	0	2.363376	-6.934587	2.213255
108	1	0	1.073960	-6.368842	6.260698
109	6	0	-1.558075	6.724209	4.181697
110	6	0	-2.516036	6.507289	3.197758
111	6	0	-3.607630	5.633815	3.406953
112	6	0	-3.742987	4.991965	4.674668
113	6	0	-2.863960	5.350989	5.739565
114	6	0	-1.799122	6.189117	5.473388
115	1	0	-2.363408	6.934585	2.213355
116	6	0	-4.503618	5.258753	2.324922
117	6	0	-4.698844	3.938142	4.809863
118	6	0	-5.376535	3.454845	3.727357
119	6	0	-5.270939	4.075337	2.443607
120	1	0	-6.047534	2.613332	3.857931
121	6	0	-4.618489	6.014802	1.123033
122	6	0	-5.396672	5.589521	0.077404

123	6	0	-6.016461	4.305613	0.089740
124	6	0	-5.908287	3.523817	1.269638
125	1	0	-4.107552	6.968380	1.043906
126	1	0	-5.465245	6.209362	-0.809565
127	6	0	-6.651979	3.746318	-1.084002
128	6	0	-6.341131	2.166733	1.226510
129	6	0	-6.749210	1.590025	0.054093
130	6	0	-6.889933	2.352543	-1.139471
131	1	0	-6.263312	1.549702	2.114063
132	1	0	-7.007939	0.536996	0.045490
133	6	0	-7.049738	4.544077	-2.202887
134	6	0	-7.606705	3.984115	-3.319974
135	6	0	-7.676948	2.565223	-3.484716
136	6	0	-7.253730	1.740014	-2.403282
137	1	0	-6.960251	5.623374	-2.144485
138	6	0	-8.074182	1.950881	-4.712514
139	6	0	-7.056945	0.360250	-2.631758
140	6	0	-7.289268	-0.217487	-3.873540
141	6	0	-7.879724	0.592331	-4.879000
142	1	0	-6.567524	-0.227140	-1.864712
143	1	0	-8.152369	0.140504	-5.828524
144	1	0	-1.073928	6.368706	6.260759
145	1	0	3.470724	4.840655	-5.777859
146	1	0	-4.629241	-7.946792	4.122795
147	1	0	-3.470832	-4.840701	-5.777891
148	1	0	-7.946837	4.629275	-4.122780
149	1	0	-4.840699	3.470635	5.777879
150	1	0	7.946956	-4.629202	-4.122778
151	1	0	4.840744	-3.470782	5.777863
152	6	0	-2.777550	-8.641927	5.842167
153	1	0	-3.493027	-7.907052	6.230287
154	1	0	-3.356285	-9.516032	5.523118
155	1	0	-2.138205	-8.950272	6.673056
156	6	0	-4.754434	-3.027221	-7.117747
157	1	0	-3.667309	-2.884709	-7.110546
158	1	0	-4.945433	-4.025700	-7.527076
159	1	0	-5.178380	-2.296647	-7.811037
160	6	0	-3.027155	4.754145	7.117726
161	1	0	-4.025624	4.945105	7.527099
162	1	0	-2.884626	3.667023	7.110463
163	1	0	-2.296567	5.178069	7.811014
164	6	0	-8.641947	2.777603	-5.842192
165	1	0	-9.516043	3.356354	-5.523150
166	1	0	-7.907053	3.493067	-6.230301

167	1	0	-8.950297	2.138270	-6.673089
168	1	0	4.629223	7.946892	4.122769
169	6	0	2.777539	8.641972	5.842172
170	1	0	3.493042	7.907115	6.230281
171	1	0	3.356245	9.516091	5.523113
172	1	0	2.138201	8.950301	6.673073
173	6	0	4.754328	3.027178	-7.117711
174	1	0	3.667206	2.884649	-7.110479
175	1	0	4.945299	4.025653	-7.527067
176	1	0	5.178269	2.296598	-7.810998
177	6	0	8.642098	-2.777506	-5.842149
178	1	0	9.516197	-3.356242	-5.523086
179	1	0	7.907228	-3.492983	-6.230284
180	1	0	8.950457	-2.138161	-6.673032
181	6	0	3.027206	-4.754316	7.117690
182	1	0	4.025679	-4.945297	7.527043
183	1	0	2.884685	-3.667194	7.110464
184	1	0	2.296624	-5.178257	7.810973

Supplementary References

1. Fleming, R. H., Quina, F. H. & Hammond, G. S. Mechanisms of photochemical reactions in solution. LXXXI. Photocyclization of 1,8-divinylnaphthalene. A new method for determining the multiplicity of excited state intermediates. *J. Am. Chem. Soc.* **96,** 7738-7741 (1974).
2. Nate, H., Sekine, Y., Honma, Y., Nakai, H., Wada, H., Takeda, M., Yabana, H. & Nagao, T. Synthesis of 2-phenylthiazolidine derivatives as cardiotonic agents. I. 2-Phenylthiazolidine-3-thiocarboxamides. *Chem. Pharm. Bull.* **35,** 1953-1968 (1987).
3. Frisch, M. J., Trucks, G. W., Schlegel, H. B., Scuseria, G. E., Robb, M. A., Cheeseman, J. R., Scalmani, G., Barone, V., Petersson, G. A., Nakatsuji, H., Li, X., Caricato, M., Marenich, A. V., Bloino, J., Janesko, B. G., Gomperts, R., Mennucci, B., Hratchian, H. P., Ortiz, J. V., Izmaylov, A. F., Sonnenberg, J. L., Williams-Young, D., Ding, F., Lipparini, F., Egidi, F., Goings, J., Peng, B., Petrone, A., Henderson, T., Ranasinghe, D., Zakrzewski, V. G., Gao, J., Rega, N., Zheng, G., Liang, W., Hada, M., Ehara, M., Toyota, K., Fukuda, R., Hasegawa, J., Ishida, M., Nakajima, T., Honda, Y., Kitao, O., Nakai, H., Vreven, T., Throssell, K., Montgomery, Jr. J. A., Peralta, J. E., Ogliaro, F., Bearpark, M. J., Heyd, J. J., Brothers, E. N., Kudin, K. N., Staroverov, V. N., Keith, T. A., Kobayashi, R., Normand, J., Raghavachari, K., Rendell, A. P., Burant, J. C., Iyengar, S. S., Tomasi, J., Cossi, M., Millam, J. M., Klene, M., Adamo, C., Cammi, R., Ochterski, J. W., Martin, R. L., Morokuma, K., Farkas, O., Foresman, J. B. & Fox, D. J. *Gaussian 16, Revision B.01* (Gaussian,

Inc., 2016).

4. Becke, A. D. Density-functional exchange-energy approximation with correct asymptotic behavior. *Phys. Rev. A* **38,** 3098-3100 (1988).

5. Becke, A. D. Density-functional thermochemistry. III. The role of exact exchange. *J. Chem. Phys.* **98,** 5648-5652 (1993).

6. Lee, C., Yang, W. & Parr, R. G. Development of the Colle-Salvetti correlation-energy formula into a functional of the electron density. *Phys. Rev. B* **37,** 785-789 (1988).

7. Ditchfield, R., Hehre, W. J. & Pople, J. A. Self-consistent molecular-orbital methods. IX. An extended Gaussian-type basis for molecular-orbital studies of organic molecules. *J. Chem. Phys.* **54,** 724-728 (1971).

8. Hehre, W. J., Ditchfield, R. & Pople, J. A. Self-consistent molecular orbital methods. XII. Further extentions of Gaussian-type basis sets for use in molecular orbital studies of organic molecules. *J. Chem. Phys.* **56,** 2257-2261 (1972).

9. Hariharan, P. C. & Pople, J. A. Accuracy of AH_n equilibrium geometries by single determinant molecular orbital theory. *Mol. Phys.* **27,** 209-214 (1974).

10. Gordon, M. S. The isomers of silacyclopropane. *Chem. Phys. Lett.* **76,** 163-168 (1980).

11. Hariharan, P. C. & Pople, J. A. The influence of polarization functions on molecular orbital hydrogenation energies. *Theor. Chim. Acta.* **28,** 213-222 (1973).

12 Bauernschmitt, R. & Ahlrichs, R. Treatment of electronic excitations within the adiabatic approximation of time dependent density functional theory. *Chem. Phys. Lett.* **256,** 454-464 (1996).

13. Hitosugi, S., Nakanishi, W., Yamasaki, T. & Isobe, H. Bottom-up synthesis of finite models of helical (*n*,*m*)-single-wall carbon nanotubes. *Nat. Commun.* **2,** 492 (2011).

14. Matsuno, T., Kamata, S., Hitosugi, S. & Isobe, H. Bottom-up synthesis and structures of π-lengthened tubular macrocycles. *Chem. Sci.* **4,** 3179-3183 (2013).

15. Kogashi, K., Matsuno, T., Sato, S. & Isobe, H. Narrowing segments of helical carbon nanotubes with curved aromatic panels. *Angew. Chem. Int. Ed.* **58,** 7385-7389 (2019).
